# A hybrid PCA-ICA and multi-level feature scaling framework with bidirectional LSTM-GRU architecture improves multivariate time series forecasting accuracy

**DOI:** 10.1038/s41598-026-51868-2

**Published:** 2026-05-18

**Authors:** Yuvaraja Boddu, A. Manimaran, Jayanth Talabathula, M. Sucharitha

**Affiliations:** 1 Department of Mathematics, School of Advanced Sciences, VIT-AP University, Amaravati, Andhra Prdaesh 522241 India; 2 School of Electronics Engineering, VIT-AP University, Amaravati, Andhra Pradesh 522241 India

**Keywords:** Multivariate time series forecasting, Hybrid feature scaling, PCA-ICA dimensionality reduction, Bi-LSTM-GRU neural network, Component-wise inverse projection, Engineering, Mathematics and computing

## Abstract

Precise multivariate time series (MTS) forecasting, particularly in atmospheric applications like air quality monitoring, is still a challenging task because of high dimensionality, temporal correlations, and non-stationary interactions between features. The classical methods such as Auto Regressive Integrated Moving Average) ARIMA and isolated Long Short-Term Memory (LSTM) are likely to fail to capture nonlinear relationships and are highly sensitive to the scale of features, and Principal Component Analysis (PCA) based dimensionality reduction is likely to result in information loss. To mitigate these constraints, we introduce PIHS-Bi-LSTM-GRU, a deep learning hybrid model that combines PCA-ICA-based reduction of dimensions, multi-level hybrid scaling of features, and an improved Bidirectional LSTM- Gated Recurrent Unit (GRU) architecture with dual-layer normalization and dropout. Our approach begins by taking a weighted ensemble of Min-Max, Z-Score, and Robust scalers for stabilizing heterogeneous distributions of features. PCA is used to alleviate redundancy, followed by Independent Component Analysis (ICA) to yield statistically independent latent signals. The deep learning model subsequently learns temporal patterns from the transformed sequences. A new component-wise inverse transformation mechanism provides exact reconstruction in the original feature space. Comprehensive evaluation on actual air quality data shows that the proposed model considerably outperforms baseline methods in Mean Absolute Error (MAE), Root Mean Square Error (RMSE), Mean Absolute Percentage Error (MAPE), and $R^{2}$ on all features. The findings verify the effectiveness of the framework in picking up on intricate temporal-spatial relations and enhancing predictive reliability under multivariate prediction situations.

## Introduction

Accurate forecasting of MTS has gained relevance in most real-life scenarios including air quality forecasting, energy demand forecasting, climate forecasting, healthcare analytics, and financial market forecasting^1,2^. In contrast to univariate forecasting, MTS is associated with a number of interdependent variables that develop concurrently and, thus, the modeling process becomes much more complicated. Such datasets are usually nonlinear in time, cross-variable, and dynamic in statistical variations, which are extremely difficult to address using the conventional forecasting techniques^3,4^. Moreover, the contemporary real-life data are usually very high-dimensional and noisy, which complicates the learning process even more. Recent studies have thus indicated the necessity of enhanced hybrid learning models that are able to derive useful temporal representations of rich multivariate data^5^.

High dimensionality and redundancy of variables is one of the most significant challenges of the MTS forecasting. Most of the features are highly correlated or collinear, and this may deteriorate the performance of the model and raise the complexity of computation. PCA and ICA are dimensionality reduction methods that have been used extensively to overcome this obstacle because they are able to extract succinct yet informative representations of high-dimensional data^6^. Moreover, multivariate time series data may be non-stationary, meaning that such statistical properties as the mean and variance change with time. These differences cause the extra complexity of learning and can diminish the forecasting accuracy when not managed. The fact that the scales of features in each environmental or financial variable are heterogeneous also implies that there is a need to have strong preprocessing methods which would stabilise the learning process.

Historically, time series analysis has been based on the use of the traditional statistical forecasting techniques as Auto Regressive Integrated Moving Average (ARIMA), Vector Auto Regression (VAR), and Holt-Winters. Nevertheless, the approaches are based on powerful assumptions like linearity and stationarity and this restricts their application in multivariate settings^4^. Specifically, ARIMA models are poor at modeling nonlinear dependencies whereas VAR models experience parameter explosion as more variables are added. Such techniques are also heavily reliant on manual pre-processing steps like lag selection and differencing, making them less scalable and less automatable.

To address these weaknesses, deep learning models, specifically recurrent neural networks (RNNs) and their subtypes including LSTM and GRU have become of great interest in time series forecasting tasks^7^. These models can automatically acquire temporal dependence and complicated nonlinear connections out of sequential data. Application to water demand forecasting, stock market prediction and air quality analysis are some of the domains where hybrid deep learning architectures have been applied successfully. Nevertheless, even deep learning models that are directly trained on high-dimensional multivariate-value inputs face the risk of overfitting and unstable training behavior. Besides, straightforward normalization methods do not typically provide sufficient support to heterogeneous distributions of features inherent in real-world data.

Even though LSTM and GRU networks have proven to be effective in capturing temporal dependencies, they can still have difficulties in capturing multivariate complex contextual relationships. Recent works have thus examined the next generation architectures of graph neural networks, transformer, and spatiotemporal learning to enhance forecast accuracy^8–10^. Such models improve the power of spatial and temporal capture of interaction between variables but at the cost of additional computational complexity. Also, more recent models like GINAR+, MetaGNSDformer and SDR-GNN have gone on to showcase the promise of hybrid learning paradigms in facing incomplete or dynamic multivariate data conditions^11,12^. Although these developments have been made, a single framework that combines effective integration of powerful preprocessing, dimensionality transformation, and hybrid temporal modeling is still required.

Driven by these issues, this paper presents a new PIHS-Bi-LSTM-GRU hybrid forecasting model that is to be developed as a precise and understandable multivariate time series prediction. The suggested model incorporates multi-level hybrid scaling, multi-stage dimensionality reduction with PCA and ICA, and a hybrid Bidirectional LSTM-GRU temporal learning model. This unified model is supposed to be more feature-representative, more capable of modeling temporal dependencies, and more stable during training in multivariate complex environments. Also, the framework has a component-wise inverse reconstruction mechanism which allows to transform the predicted values to the original feature space precisely to enhance interpretability and practical use.

The essence of the suggested method is that the PIHS multi-level hybrid scaling strategy is a combination of the pros of Min-Max normalization, Z-score standardization, and Robust scaling. This scaling method of hybrid is used to provide equal distributions of features and enhance training stability in heterogeneous variables. Combining sophisticated preprocessing, dimensionality decomposition, and hybrid recurrent learning into a single architecture, the proposed framework offers a useful solution to the problems of the modern multivariate time series forecasting.

Using weighted fusion, the model can adaptively normalize the heterogeneous features, maintain relative relations, and avoid outlier impacts. A similar approach is especially important in actual environmental data sets where variables such as wind speed and snow depth have strongly correlated, highly skewed distributions. After normalization, a two-stage dimensionality reduction strategy is utilized. Initially, the input space is compressed by PCA, which preserves the principal axes of variation, eliminating redundancy and noise. Then, Fast Fast ICA is applied to PCA-reduced data to obtain statistically independent latent features, which are more appropriate for sequence modelling and tend to represent useful real-world signals.

These decorrelated, compressed feature sequences are then fed into a deep learning network that benefits from the representational capacity of Bidirectional LSTM and the computational simplicity of GRU. The Bi-LSTM-GRU hybrid architecture is extended with Layer Normalization and Dropout at several points to improve training stability, speed up convergence, and prevent overfitting. The bidirectional aspect allows the model to learn both past and future context, which is especially useful for applications where causal and feedback relations among features exist. The addition of GRU maximally improves memory efficiency and training time without compromising accuracy.

One of the most groundbreaking parts of the PIHS-Bi-LSTM-GRU framework is its component-wise inverse projection pipeline, which enables projected forecasted outputs in ICA space to be mapped back into the original feature dimensions. This is done through sequential use of inverse ICA, inverse PCA, and an analytically calculated inversion of the hybrid scaling function. This reconstruction process retains the structural integrity of the predictions and ensures that the output is directly interpretable and actionable in real-world scenarios. As opposed to black-box models, PIHS-Bi-LSTM-GRU retains an open connection between its predictions and input variables, thus providing higher trustworthiness and interpretability.

PCA is effective in reducing dimensionality by capturing the directions of maximum variance and removing redundant correlations among variables, while ICA focuses on extracting statistically independent latent components, which helps in separating mixed signals and reducing hidden dependencies in multivariate data. By combining these two techniques, the proposed PICA framework first performs variance-based compression through PCA and subsequently applies ICA to obtain independent representations, resulting in a more informative and noise-resistant feature space for forecasting.

### Contribution and novelty of the work

- A robust Adaptive Multi-Level Hybrid Scaling (MLHS) framework is proposed to normalize heterogeneous multivariate features through a weighted integration of Min-Max, Z-score, and robust scaling techniques, ensuring statistical consistency and reduced sensitivity to outliers.
- A novel dual-stage PICA dimensionality reduction approach is introduced, combining PCA for variance preservation and ICA for independence maximization, resulting in compact, de-correlated, and information-rich feature representations for efficient multivariate sequence learning.
- A hybrid Bi-LSTM-GRU architecture with layer normalization and dropout is developed to effectively capture both local and global temporal dependencies, including forward and backward dynamics, while improving convergence stability and mitigating overfitting.
- An invertible component-wise projection mechanism is designed to map latent predictions back to the original feature space through a structured inverse pipeline (ICA $\rightarrow$ PCA $\rightarrow$ scaling), enabling interpretability and seamless integration into real-world decision-support systems.

Overall, this research work makes the following fundamental contributions. It introduces a new high-variability adaptive hybrid feature scaling method. It suggests a two-stage PCA-ICA decomposition approach for robust dimensionality reduction and feature independence. It develops a deep hybrid sequence model that combines bidirectional LSTM and GRU units with dual layer normalization and dropout. It constructs a mathematically motivated inversion procedure that projects latent forecasts back to the data space of origin at high fidelity. Finally, through large-scale experimentation on real-world air pollution datasets, it shows that the PIHS-Bi-LSTM-GRU model is superior to traditional statistical models, typical LSTM/GRU networks, and even PCA-augmented deep learning approaches in predictive accuracy, interpretability, and computation cost.

The issue of air pollution forecasting has become an important research issue because of its direct effects to the population health, environmental sustainability and urban planning. The precise forecasting of the pollutants including PM2.5 is very difficult due to the nonlinear, non-steady, and multivariate characteristics of atmospheric data, in which there exists numerous interdependences between meteorological and anthropogenic variables. Recent research work has pointed out that traditional statistical models are usually inadequate to represent these more complex temporal dependencies, and thus their predictive power when used in practice is restricted. To overcome the above challenges, previous research can be classified into three streams. First, the conventional methods like ARIMA and its derivatives are linear in nature, and they cannot be used to determine large nonlinear dynamics. Second, more sophisticated neural network models, such as LSTM, Bi-LSTM and GRU, have shown better performance by exploiting temporal dependencies, but tend to be susceptible to high-dimensional feature overlaps and lack interpretation. Third, the recent developments are oriented towards hybrid and transformer-based architectures, including PatchTST and iTransformer, which apply attention mechanisms and long-range dependency modelling, but remain challenging due to feature noise and computational complexity. Although these have been made, little effort has been put in the integration of complementary techniques of dimensionality reduction, including PCA and ICA, into a single framework to achieve simultaneous redundancy and statistical independence of multivariate inputs. This is where PICA-based hybrid model is proposed, and the purpose of the hybrid is to increase the representation of features and the accuracy of forecasting by balancing the orthogonal variance preservation with higher statistical independence to give a more robust solution to multivariate air pollution forecasting.

This background sets the stage for a more thorough discussion of the suggested architecture, its theoretical foundations, its implementation specifics, and empirical testing. The rest of this paper is structured such that the following section provides an overview of related work on MTS forecasting, feature scaling, dimensionality reduction, and hybrid deep learning architectures. This is succeeded by a detailed methodology section outlining the PIHS-Bi-LSTM-GRU architecture. Following sections describe the experimental setup, dataset overview, performance metrics, and result interpretation. The last section summarizes the paper and discusses future research directions to apply the model in various MTS domains.

The originality of the suggested PICA-Bi-LSTM-GRU architecture has been placed more clearly in opposition to the recent hybrid and Transformer-based ones. The current methods, such as PatchTST and iTransformer variants of Transformer, assume as input raw or linearly embedded values and utilize attention mechanisms to implicitly learn feature relationships, which can be inefficient in managing high-dimensional correlated multivariate time series data that is noisy and correlated. Furthermore, earlier hybrid models generally combine dimensionality reduction or deep learning modules individually, without defining a structured pipeline to jointly optimize feature decorrelation, statistical independence, and temporal modeling. The current gap is bridged by the suggested method proposing a single architecture that first removes redundancy through PCA, then finds independent latent signals through ICA and finally uses a hybrid Bi-LSTMGRU network to efficiently learn over time. Such sequential integration explicitly aims to address three major limitations of previous research: (i) no noise-resilient feature transformation, (ii) insufficient use of feature interdependencies, and (iii) poor temporal representation when heterogeneous data are available. As well, a comparative architectural scheme has been included to explicitly show how the proposed pipeline compares to Transformer and hybrid models of the baseline, thus reinforcing the clarity of its incremental contribution and design thinking.

## Relative work

MTS prediction has emerged as an essential area of research because it has a wide range of applications in environmental surveillance, stock markets, energy predictions, and health analytics. Temporal dependencies, high dimensions, and complex relationships among the variables complicate the data, making standard model-based methods unsuitable for practical utilization. Considering these challenges, a multitude of machine learning and deep learning approaches have been developed to enhance the predictive validity, scalability, and interpretability of forecasting models. The current section provides a comprehensive review of the literature, placing the journey from conventional methods towards hybrid deep learning models in perspective and pointing out the contributions and shortcomings of each.

Early MTS forecast models were essentially statistical, based on the stationarity and linearity assumptions. Methods like ARIMA and VAR models were overarching initial research but ultimately proved inadequate in handling intricate and non-linear phenomena characteristic of MTS data^1^. Such models generally had inherent problems of overfitting, unsalability with growing dimensions, and failure to capture long-range dependencies^13^. Consequently, the transition towards data-driven methods became imminent. The invention of deep learning revolutionized time series analysis by making it possible for models to learn temporal information directly from unprocessed data. RNNs and specifically LSTM networks proved to be highly successful in sequence modelling because of their memory-based architecture that can learn long-term dependencies^7^. However, LSTM networks are computationally costly and overfit on high-dimensional data. To overcome these challenges, researchers started investigating combinations of deep learning architectures and dimensionality reduction methods.

Both ICA and PCA are heavily utilized for dimensionality reduction and elimination of redundancy in multivariate data^14^. PCA finds orthogonal directions that have the most variance, whereas ICA separates statistically independent components. Sarikoç and Celik suggested a hybrid PCA-ICA-LSTM model for predicting the S and P 500 index, proving that their combination was superior in terms of feature extraction and prediction accuracy^3^. Xu et al. combined ICA and PCA with relevance vector machines for multivariate process monitoring, with a focus on the need for prior de-correlation of the components prior to modeling^4^. Deep architectures have been coupled with these decomposition methods in numerous studies. Uçkan used PCA with LSTM in Turkish stock market prediction and reported significant improvements in prediction accuracy^2^. Liu and Lai introduced a PCA-GRU-LSTM model that adequately maintained a balance between feature reduction and temporal learning and is appropriate for finance use^6^. In the field of environmental information, Du et al. proposed a hybrid deep learning architecture for air quality prediction that utilized LSTM in conjunction with CNN-based feature extraction to model spatial-temporal relationships^15^. Their system surpassed traditional statistical models, especially on noisy and heterogeneous data sets.

Hybrid neural network architectures further improved temporal learning by combining the advantages of various RNN types. For example, combining Bidirectional LSTM (Bi-LSTM) and GRU has been reported to enhance forecasting accuracy through the capture of both past and future contexts. Li et al. introduced a Bi-GRU model optimized using Sparrow Search Algorithm (SSA) for production forecasting, showing enhanced convergence and accuracy^16^. Singh et al. suggested a CNN-GRU-LSTM model for traffic forecasting, reporting that hybrid models can perform better than single models through complementary strengths^17^. Attention mechanisms also play a central role in boosting deep learning models for MTS. Zhou et al. proposed an attention-based CNN-LSTM model for urban water demand forecasting, allowing the model to concentrate on the most important time steps and features^18^. In the same vein, Boddu and Manimaran proposed the QPCA-LSTM model, which pairs quartet-based PCA with LSTM to achieve maximal forecasting accuracy^19^. Their findings showed that coupling attention with dimensionality reduction can dramatically improve interpretability and accuracy.

One interesting advancement in this direction is the implementation of hybrid scaling techniques to pre-process MTS data for learning more effectively. Typical scaling techniques such as Min-Max or Z-Score prove to be inadequate if applied standalone on heterogeneous features. Recent work has embraced hybrid normalization to counter this limitation. For instance, this current work proposes a weighted combination of Min-Max Scaler, Standard Scaler, and Robust Scaler, allowing the model to learn to adapt to different scales and outliers. This idea is supported by previous research by Pradhan and Panigrahi, where they reported that incorrect scaling might result in the deterioration of performance in AQI prediction tasks^20^.

There has also been a recent focus on the need for interpretable and invertible models. Reconstruction methods in components have gained significance in converting latent predictions back to the original feature space. This is highly applicable in environmental and financial contexts where actionable information relies on domain-specific measures. Chen et al. spoke of component decomposition in hyperspectral data and its application to anomaly detection, establishing precedent for its applicability in time series forecasting^21^. The PIHS-Bi-LSTMGRU model takes this as a starting point by allowing for inverse ICA $\rightarrow$ PCA $\rightarrow$ hybrid scaling transformations, hence increasing interpretability.

Temporal modelling has also been innovated using transformer-based and graph-based architectures. Wu et al. designed a graph neural network (GNN) for MTS prediction, representing variables as nodes in a graph to learn their interdependency^22^. Zheng and Hu constructed a temporal change information boosting model, which was effective in dynamic settings^23^. However, such models tend to be dependent on large-scale datasets and are computationally expensive. On the other hand, such hybrid RNN models as PIHS-Bi-LSTMGRU provide a trade-off between performance and complexity.

Hybrid deep learning architectures have also succeeded in energy and finance applications. Dalal et al. introduced TLIA, a hybrid of LSTM and artificial neural networks designed for unstable energy markets, which showcases better flexibility^24^. Zhang et al. combined CEEMD and LSTM for forecasting financial time series, showcasing the superiority of the decomposition-based models^25^. The PCA-ICA basis of the proposed approach follows this trend, providing increased forecasting stability.

As far as practical application is concerned, air pollution level forecasting has been a major challenge. Subramaniam et al. surveyed AI models for air quality and health prediction and mentioned that such models that are hybrid combinations of decomposition, scaling, and deep learning tend to provide the highest accuracy^26^. Samal et al. also showed the efficacy of multi-output neural networks for Spatio-temporal forecasting of air pollution^27^. These studies highlight the significance of feature interaction modelling and multivariate learning, both of which are at the core of the PIHS-Bi-LSTMGRU framework. The model’s robustness also mirrors trends in recent metaheuristic-included architectures. Boddu et al. introduced iterative dual-metaheuristic models and temporal attention-based approaches for time series prediction. These models became more efficient and accurate, especially in high-dimensional scenarios^28,29^. Although this paper does not use metaheuristics, it inherits similar iterative concepts through inverse transformations and control of dimensionality.

Further developments have investigated the use of attention, hybrid models, and new temporal representations to improve predictive ability. Boddu and Manimaran introduced hybrid models like TVE-MTAN^30^, TGAMTSA^31^, and IFCAR-SHL Net^32^, which integrated attention models with dimensionality reduction as well as multi-level neural architectures for better multivariate time series analysis.

Mathonsi and van Zyl introduced a statistical-deep learning combination for mortality modelling, focusing on reconciling statistical interpretability with deep feature learning^33^. Liu et al. provided a secondary decomposition approach combined with deep models for PM2.5 forecasting, presenting improved short-term prediction ability^34^. Xing et al. applied LSTM networks across climate regions for solar radiation forecasting, highlighting model flexibility across domains^35^. Pavlicko et al. used neural networks for national energy prediction, demonstrating actual-world applications of such hybrid MTS models^36^. Patil et al. also presented upcoming hybrid learning approaches from the ICICBDA 2024 conference, highlighting the wide range of intelligent forecasting structures’ applications^37^.

The advanced recent developments of MTS forecasting have been more inclined at incorporating spatiotemporal learning, transformer systems and graph-based representations to represent the intricate interdependence among variables and time. As an example, MGSFformer model introduced by Chengqing Yu et al.^8^ presents a multi-granularity spatiotemporal fusion transformer that can effectively learn hierarchical time dynamics in predicting air quality. On the same note, the GINAR+ framework^5^ responds to the missing value and robustness concerns with a better end to end forecasting behaviour in the real-world multivariate scenarios. Simultaneously, highly developed transformer-based architectures like Meta GNSDformer^11^ introduce meta-learning and frequency-sensitive attention systems to help in boosting prediction accuracy in non-stationary situations. Graph neural network (GNN)-based solutions have also become eminent to model the relationships among variables. An effective spatial and temporal correlations across many sites are well represented by the DEST-GNN model^9^ and the parallel multi-scale dynamic GNN described by Mingjie Hou et al.^10^, making them appropriate in complex forecasting problems such as energy and environmental systems. SDR-GNN^12^ also uses spectral domain reconstruction to deal with the multimodal data that are incomplete and enhances robustness.

Although these models are very successful, they can be characterized by great computational complexity and preprocessing of data can be associated with significant costs. The suggested PIHS-Bi-LSTM-GRU model, on the contrary, takes a more computationally efficient and effective hybrid approach, which combines multi-level scaling, dimensionality reduction through PCA-ICA, and hybrid recurrent modeling. Compared to transformer and GNN-based models, the proposed solution is more concerned with the enhancement of feature representations and temporal learning synergy, which precondition its appropriateness to high-dimensional and noisy multivariate data and allows it to be scaled to practice and interpreted.

While the current models like LSTM and GRU are impressive in learning long-term temporal relationships, they tend to be plagued by overfitting and poor interpretability, particularly when used for direct application on high-dimensional multivariate inputs. The PCA-LSTM model does solve dimensionality reduction but involves orthogonality instead of independence, which tends to hide underlying causal mechanisms and non-Gaussian processes. ICA-based models, in contrast, enhance signal separation but are generally noise-sensitive and lack variance-preserving efficiency. Additionally, Transformer-based models, although effective in capturing global temporal context, demonstrate quadratic time complexity (O($\textrm{T}^{2}$)), which constrains their use in real-time or long-sequence tasks. Perhaps most importantly, none of the models utilize an invertible projection mechanism, complicating efforts to back-project latent predictions into expressive, real-world feature spaces. These constraints point to the necessity for a single model that combines adaptive scaling, statistical decomposition, temporal modelling, and inverse transformation realized by the proposed PIHS-Bi-LSTM-GRU framework.

In short, the literature documents a distinct line of progression from simple statistical modelling to more and more sophisticated hybrid structures that include dimensionality reduction, normalisation, deep temporal modelling, and interpretability mechanisms. The new PIHS-Bi-LSTM-GRU architecture continues this line of progression by incorporating hybrid scaling, PCA-ICA decomposition, Bi-LSTM-GRU modelling, and inverse transformation to overcome persistent MTS forecasting challenges at large in one single approach. By a delicate combination of innovation and computational tractability, it is aligned with current research and makes new contributions to the area.

The structure of the paper is set up to methodically demonstrate the creation and verification of the suggested forecasting framework. A thorough analysis of related literature is provided in Section 2, which also identifies current gaps in multivariate time series forecasting and dimensionality reduction methods. The integration of PICA with Multi-Level Hybrid Scaling and a Bi-LSTM-GRU model improved with normalization and dropout are highlighted in Section 3, which provides specifics on the suggested methodology. The experimental dataset, feature selection, preprocessing techniques, and the justification for using environmental time series data are all covered in Section 4. The experimental findings are shown in Section 5, where standard metrics are used to compare the performance of the suggested model to many baseline models. The significance of these findings is covered in Section 6, where they are backed up by results from Diebold-Mariano (DM) tests and residual analysis that demonstrate statistical superiority. Section 7 wraps up the study by highlighting the main conclusions and suggesting future lines of inquiry to improve forecasting precision and practicality.

## Proposed PIHS-Bi-LSTM-GRU framework

The suggested forecasting model iteratively combines dimensionality reduction and deep learning to improve multivariate time series data prediction accuracy. First, the raw data is put through multi-level hybrid scaling, which uses a weighted ensemble of Min-Max, Z-Score, and Robust scalers to efficiently normalize features while retaining distributional properties and lessening outlier influences. After normalization, the dimensionality is reduced in a two-step process: Principal Component Analysis (PCA) retains the maximum variance in orthogonal directions, and Independent Component Analysis (ICA) subsequently breaks down the signals into statistically independent components, thereby allowing for more efficient and effective representation of the underlying patterns.

The data thus obtained is then restructured into supervised learning sequences, allowing temporal modelling through a hybrid Bi-LSTM-GRU architecture. The bidirectional LSTM incorporates contextual data from both previous and subsequent time steps, while the GRU optimizes short-term dynamics. Layer Normalization stabilizes training, and Dropout prevents overfitting, resulting in better generalization. Lastly, predictions are projected back into the original feature space via an inverse transformation pipeline, maintaining interpretability. The overall architecture exhibits higher predictive accuracy, for which it is particularly appropriate for high-dimensional, complex datasets like those encountered in financial analysis and environmental monitoring.

**Fig. 1:**
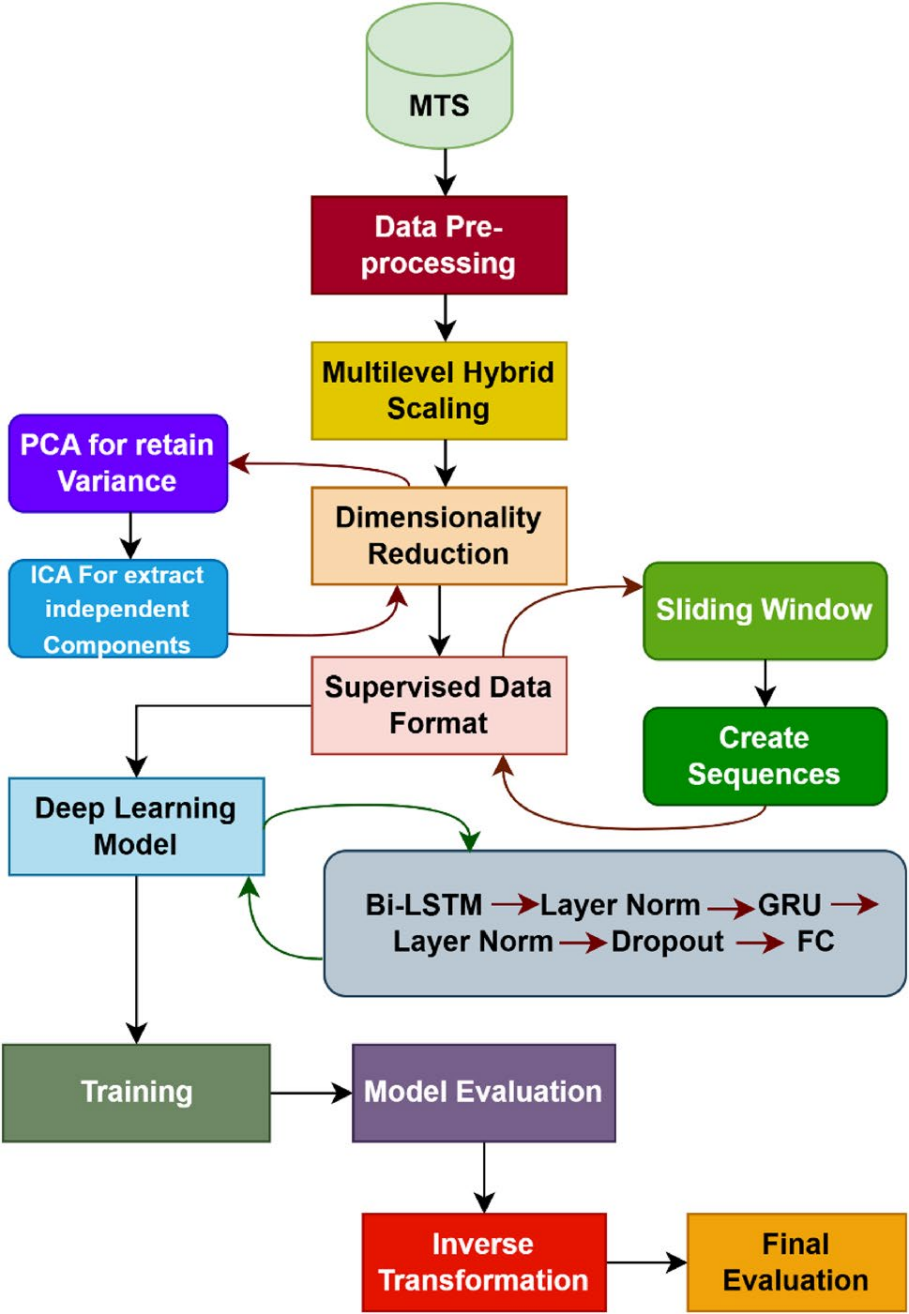
Proposed PIHS-Bi-LSTM-GRU methodology.

The provided Figure 1 outlines the end-to-end structure of the envisioned PIHS-Bi-LSTM-GRU prediction framework, incorporating a structured sequence from raw MTS collection to actionable prediction results. First, the pipeline performs intensive data preprocessing to resolve inconsistencies and structure readiness, after which it applies a multilevel hybrid scaling approach that consolidates heterogenous feature distributions through synergistic use of Min-Max, Z-Score, and Robust scaling. Then, a two-stage dimensionality reduction procedure PCA for retaining variance and ICA for isolating statistically independent underlying factors - yields a compact, decorrelated feature space favourable for effective modeling. Utilizing a sliding window approach, the minimized data is converted into supervised learning sequences, which are subjected to processing via a hybrid deep learning model that combines bidirectional LSTM for end-to-end temporal dependency capture, GRU for efficiency in computation refinement, and carefully positioned layer normalization and dropout layers for stabilized training and strong generalization. The fully connected output layer provides predictions that go through a mathematically accurate inverse transformation pipeline (inverse ICA $\rightarrow$ inverse PCA $\rightarrow$ inverse scaling) for reconstruction in the target feature domain for semantic consistency. Final evaluation integrates model performance evaluation, affirming both prediction fidelity and operational interpretability across noisy, complex, and high-dimensional time series settings.

### Multi-level hybrid scaling

In MTS prediction, the preprocessing phase plays a critical role in determining the quality of the subsequent learning. Standard normalization methods albeit effective in single-handed ways tend not to preserve both the scale-sensitive dynamics and statistical heterogeneity of features with heterogeneous distributions, skewness, or outlier sensitivity. To improve on this, we introduce an innovative Multi-Level Hybrid Scaling (MLHS) approach. This method combines the robustness of three popular scalers: Min-Max Scaler, Z-Score Standard Scaler, and Robust Scaler, into one efficient, flexible, and composite representation, to achieve a strong normalization scheme specifically for varied real-world datasets.

#### Mathematical formulation

Let the input MTS be denoted as $X \in \mathbb {R}^{T \times F}$, where *T* represents the number of time steps and *F* denotes the number of features. The hybrid scaled representation $X_{\text {scaled}}$ is defined as

1 $$\begin{aligned} X_{\text {scaled}}= & \alpha \, X_{\text {Min-Max}} + \beta \, X_{\text {ZScore}} + \gamma \, X_{\text {Robust}}, \end{aligned}$$

2 $$\begin{aligned} X_{\text {Min-Max}}= & \frac{X - X_{\min }}{X_{\max } - X_{\min }}, \end{aligned}$$

3 $$\begin{aligned} X_{\text {ZScore}}= & \frac{X - \mu }{\sigma }, \end{aligned}$$

4 $$\begin{aligned} X_{\text {Robust}}= & \frac{X - \operatorname {Median}(X)}{\operatorname {IQR}(X)}. \end{aligned}$$

Let $X \in \mathbb {R}^{N \times d}$ denote the original feature matrix. The transformed feature representation $X^{*}$ is computed as:

5 $$\begin{aligned} \alpha + \beta + \gamma = 1, \quad \alpha , \beta , \gamma \in [0,1]. \end{aligned}$$

where:

- $X_{\text {min-max}}$ represents Min-Max normalized features,
- $X_{\text {z-score}}$ denotes standard score normalization,
- $X_{\text {robust}}$ corresponds to median and interquartile range-based scaling.

This formulation ensures that the resulting feature representation preserves complementary statistical properties, including bounded scaling, distribution normalization, and robustness to outliers.

$(X_{Min-Max})$ Scales each feature to [0, 1] range, preserving the local bounds. $(X_{ZScore})$ ensures global distributional standardization, useful for models sensitive to Gaussian assumptions and centers feature by subtracting the mean and scaling by standard deviation, this emphasizing global statistical consistency. $(X_{Robust})$ utilizes the median and Inter Quartile Range (IQR), making it resilient to outliers. The weights $\alpha$, $\beta$, and $\gamma$ are tunable and serve to balance the contribution of each scaler based on the data characteristics.

The suggested Multi-Level Hybrid Scaling (MLHS) algorithm is aimed to simultaneously extract and maintain several statistical characteristics present in multivariate time series data. Adding the Min-Max Scaler step guarantees that all features are scaled in a bounded [0, 1] interval, which is especially effective in stabilizing gradient-based optimization in neural networks. In parallel, the Z-Score standardization puts the data at the centre with unit variance, matching the requirement of most deep learning models to use normally distributed inputs for successful convergence. To counter the issues presented by outliers, which are often found in actual environmental or financial data, the Robust Scaler component utilizes median and interquartile range (IQR) to offer a flexible transformation. Through linearly weighted combination of these three scalers, the MLHS method eliminates the disadvantage of dependency on any one normalization method. Rather, it constructs a composite representation that maintains scale consistency, statistical distribution, and outlier distortion robustness, thus improving input data quality for downstream modelling processes.

The weights ($\alpha$, $\beta$, and $\gamma$) in the Multi-Level Hybrid Scaling (MLHS) formulation are important hyperparameters that regulate the relative contribution of each scaling algorithm Min-Max, Z-Score, and Robust scaling to the ultimate transformed data. Their proper selection has a direct consequence on the quality of the scaled representation and, in turn, on the performance of the forecasting model. A straightforward method is empirical tuning, where candidate values (e.g., 0.1, 0.3, 0.5, 0.7) are tried subject to the constraint $\alpha$+$\beta$+$\gamma$=1, and the best combination is chosen using validation metrics like RMSE. Or, one can use an adaptive weighting scheme based on entropy, in which the statistical spread (e.g., entropy, standard deviation) of features over the three scalers is measured and higher weights are given to the scaler that causes the least information loss.

A more demanding methodology is cross-validation-based optimization in which the weight coefficients are collectively tuned together with model-specific hyperparameters in a nested validation loop. For real-world settings or in scenarios when computational power is constrained, a fixed heuristic can be a good baseline for example, setting $\alpha$=0.3, $\beta$=0.4, and $\gamma$=0.3, thus slightly favouring Z-Score standardization with a balanced contribution of Min-Max and Robust scaling. This adaptable weighting approach ensures MLHS can be specially adapted to various datasets and learning environments, further enhancing its generalizability and robustness.

The MLHS approach has a strong benefit over the traditional single-scaler methods by combining the merits of Min-Max, Z-Score, and Robust scalers into a composite normalization model. Although Min-Max scaler well represents the local range of data, it is outlier sensitive. Z-Score normalization normalizes the shape of distribution by centering and scaling with respect to mean and variance but fails in the presence of extreme values. Robust scaler, by contrast, is specifically meant to deal with outliers via median and IQR-based transformation but ignores overall scale structure. MLHS addresses these separate limitations by combining all three, maintaining important statistical properties–local scaling, global distributional symmetry, and robustness-all at once. This holistic addressing of data heterogeneity dramatically improves model stability and forecasting precision, particularly in complicated multivariate time series applications.

### MLHS weight formulation

To enhance the mathematical rigor and reproducibility of the proposed MLHS mechanism, the hybrid normalization process is formally defined as a weighted convex combination of three scaling techniques: Min-Max, Z-score, and Robust scaling.

#### Weight determination strategy

The weights $(\alpha , \beta , \gamma )$ are determined using a **data-driven optimization approach** rather than fixed empirical selection. Specifically, Bayesian optimization is employed within a nested cross-validation framework to identify the optimal combination that minimizes prediction error.

The optimization problem is defined as:

6 $$\begin{aligned} (\alpha ^*, \beta ^*, \gamma ^*) = \arg \min _{\alpha +\beta +\gamma =1} \mathscr {L}(f(X^{*}), Y). \end{aligned}$$

where $\mathscr {L}(\cdot )$ denotes the loss function (RMSE/MAE), and $f(\cdot )$ represents the forecasting model.

This approach enables adaptive weighting based on dataset characteristics such as feature distribution, variance, and outlier presence, thereby improving generalization performance.

### Principal component analysis (PCA)

Principal Component Analysis (PCA) is a widely adopted linear dimensionality reduction technique aimed at identifying the directions (principal components) that capture the maximum variance in high-dimensional data. Given a standardized multivariate input matrix $X_{\text {scaled}} \in \mathbb {R}^{T \times F}$. The first step involves computing the covariance matrix:

7 $$\begin{aligned} \Sigma = \frac{1}{n} X^{T} X, \end{aligned}$$

Subsequently, eigen decomposition is applied

8 $$\begin{aligned} \Sigma = W \Lambda W^{T}, \end{aligned}$$

where *W* is the matrix of eigenvectors and $\Lambda$ is the diagonal matrix of eigenvalues representing the variance captured by each principal component. The data is then projected to a lower-dimensional subspace:

9 $$\begin{aligned} X_{\textrm{PCA}} = X_{\textrm{Scaled}} W_{\textrm{PCA}}, \quad W_{\textrm{PCA}} \in \mathbb {R}^{F \times k}. \end{aligned}$$

where *k* is selected such that the cumulative explained variance ratio exceeds 95%. This step compresses the input while retaining its essential structure, reducing redundancy and noise, and providing a compact representation for downstream modelling such as ICA and Bi-LSTM-GRU.

The combination of PCA and ICA is driven by the complementary nature of the two in multivariate feature transformation. PCA is mainly concerned with explaining the maximum variances and dimensions reduction which is projecting the original correlated variables into a smaller set of orthogonal components which explain the predominant variability in the data set. Nonetheless, PCA does not explicitly imply statistical independent ness of the extracted components. Conversely, ICA tries to discover statistically independent latent sources by reducing higher-order cross-dependence between variables. At single level of use, PCA might not eliminate correlated structures when used whereas ICA can be unstable when it is applied on high-dimensional noisy data directly. Thus, the presented PICA algorithm initially uses PCA to reduce the amount of feature space and eliminate duplicate correlations and then ICA to obtain independent latent signals. This series of changes generates a parsimonious and information-rich representation of the initial multivariate inputs, which enhances the performance of the next deep learning forecasting model.

### Independent component analysis (ICA)

Independent Component Analysis (ICA) is a statistical tool capable of converting multivariate data into maximally independent components in a non-Gaussian manner. After Principal Component Analysis (PCA), which whitens the input data by decorrelating it, a whitening operation is performed.

10 $$\begin{aligned} Z = X_{\text {PCA}} \, \Sigma ^{-\frac{1}{2}}, \end{aligned}$$

Where $\Sigma ^{-\frac{1}{2}}$ denotes the inverse square root of the PCA covariance matrix, ensuring unit variance. ICA then seeks a linear transformation

11 $$\begin{aligned} X_{\text {ICA}} = W_{\text {ICA}} \, Z. \end{aligned}$$

Where $W_{\text {ICA}}$ is calculated to optimize the components’ statistical independence. Usually, the Central Limit Theorem is used to optimize a contrast function based on non-Gaussianity, like kurtosis or negentropy. ICA efficiently distinguishes latent, independent sources embedded in the mixed signals by functioning in the whitened region. This improves model interpretability and predictive performance in time series forecasting by disentangling overlapping temporal dynamics. ICA maximizes non-Gaussianity (Kurtosis/negentropy) to extract statistically independent signals. Used after PCA to improve signal separation.

### Supervised sequence generation

Here, supervised sequence generation converts the unsupervised time series into a format that can be used to learn the temporal dependencies. From MTS data $x_t \in \mathbb {R}^{C}$ (obtained from ICA), then we form input-output pairs by applying a sliding window of size *L*. For each time step *t*, the input sequence is given by

12 $$\begin{aligned} X_t = \left[ x_{t-L},\, x_{t-L+1},\, \ldots ,\, x_{t-1} \right] , \quad y_t = x_t. \end{aligned}$$

This implies the model learns to forecast the present ICA feature vector $x_t$ based on the L previous vectors. This facilitates the network to make use of short and long-term temporal patterns over ICA-transformed features, and then reconstructed to the original feature space. Training sequence models such as Bi-LSTMGRU requires this, as it presents structured temporal input-output relations for supervised learning. *L* is the look back window size (Ex. 5) and generates input-output pairs for sequential modelling.

Algorithm 1 describes the dimensionality reduction stage, this stage preprocesses and compresses the multivariate time series data. The dataset is cleaned first by dropping categorical columns and replacing missing values. A multi-level hybrid scaling method is then applied. It uses a combination of three normalization methods Min-Max scaling, Z-score normalization, and Robust scaling to make sure the data is optimally balanced for all feature ranges, outliers, and distributions. Following scaling, two-stage dimensionality reduction is applied. Initially, Principal Component Analysis (PCA) is utilized to lower the dimensionality of features at the cost of keeping most of the significant variation in the data. This is followed by Independent Component Analysis (ICA), which is used to further convert the data into independent components, which aid in discovering special patterns or signals in the time series. The resulting diminished dataset is then transformed into deep learning appropriate sequences by generating input-output pairs with the help of a sliding time window.

**Algorithm 1:**
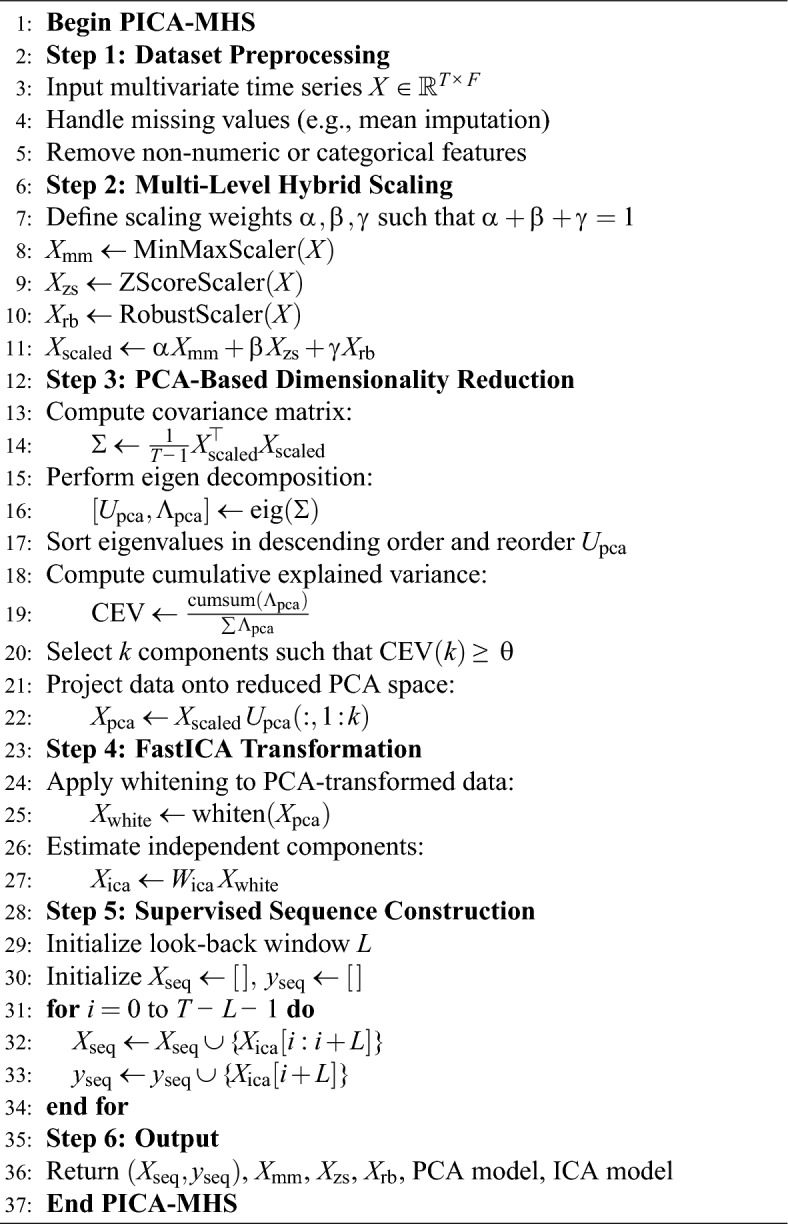
PICA with multi-level hybrid scaling framework for MTS dimensionality reduction.

### Bi-LSTM-GRU model forward pass

Next, Bi-LSTM-GRU model, the forecasting task involves learning intricate temporal dependencies from MTS transformed from PCA and ICA. To effectively model both short- and long-term dependencies, a Bidirectional LSTM layer is initially applied. It processes input sequence $x_t \in \mathbb {R}^{C}$ in both forward and backward directions, producing hidden state $h_t^{\rightarrow } \quad \text {and} \quad h_t^{\leftarrow }$. These are concatenated as shown in equation 13, capturing contextual information from previous and subsequent time steps. To normalize the output distribution and make training stable, Layer normalization is used to output of Bi-LSTM. The Layer normalization is shown in the equation 14 and here $\mu$ and $\sigma ^{2}$ are the mean and variance across features. The normalized sequence is fed through a GRU layer that captured further sequential relationships through gates controlling information flow. GRU calculates a rest gate $r_t$, updated gate $z_t$, and candidate activation $\tilde{h}_t$, updating the hidden state. Following are the process equations of Bi-LSTM (11–13), GRU (15–18) and Layer Normalization (14).

13 $$\begin{aligned} \overrightarrow{h}_t, \overrightarrow{c}_t= & \textrm{LSTM}\!\left( x_t, \overrightarrow{h}_{t-1}, \overrightarrow{c}_{t-1}\right) , \end{aligned}$$

14 $$\begin{aligned} \overleftarrow{h}_t, \overleftarrow{c}_t= & \textrm{LSTM}\!\left( x_t, \overleftarrow{h}_{t+1}, \overleftarrow{c}_{t+1}\right) , \end{aligned}$$

15 $$\begin{aligned} h_t= & \left[ \overrightarrow{h}_t , \overleftarrow{h}_t \right] , \end{aligned}$$

16 $$\begin{aligned} \textrm{LN}(h)= & \frac{h - \mu }{\sqrt{\sigma ^{2} + \varepsilon }} \cdot \gamma + \beta , \end{aligned}$$

17 $$\begin{aligned} r_t= & \sigma \!\left( W_r x_t + U_r h_{t-1}\right) \end{aligned}$$

18 $$\begin{aligned} z_t= & \sigma \!\left( W_z x_t + U_z h_{t-1}\right) , \end{aligned}$$

19 $$\begin{aligned} \tilde{h}_t= & \tanh \!\left( W_h x_t + U_h \left( r_t \odot h_{t-1}\right) \right) , \end{aligned}$$

20 $$\begin{aligned} h_t= & (1 - z_t) \odot h_{t-1} + z_t \odot \tilde{h}_t, \end{aligned}$$

21 $$\begin{aligned} \textbf{h}_t^{\text {norm}}= & \text {LN}_2 \left( \sigma \left( \text {LN}_1 \left( \textbf{h}_t \right) \right) \right) . \end{aligned}$$

This mechanism enables GRUs to selectively remember or discard old information without having complete cells like LSTMs, reducing the model’s weight and speed. There is a second layer normalization after which dropout is applied to valid overfitting. Finally, there is a fully connected (FC) layer that projects the normalized processed hidden representation to the output dimension (number of components), producing the final prediction $\tilde{y}_t$ indicate in equation 20. This pipeline guarantees the network learns deep temporal patterns while remaining regularized and stable during training.

The use of two sequential layer normalization operations is intentional and functionally motivated, rather than redundant. Specifically:

- Pre-activation normalization ($\text {LN}_1$) stabilizes the distribution of hidden representations before applying the nonlinear transformation. This reduces internal covariate shift and ensures that the activation function operates within a well-conditioned input range.
- Post-activation normalization ($\text {LN}_2$) further refines the transformed features by controlling variance amplification introduced by the nonlinear activation, thereby improving gradient flow and training stability.
- This dual normalization strategy is conceptually aligned with normalization practices in advanced deep architectures (e.g., Transformer pre-norm and post-norm variants), where multiple normalization stages enhance convergence behavior and generalization performance.

**Fig. 2:**
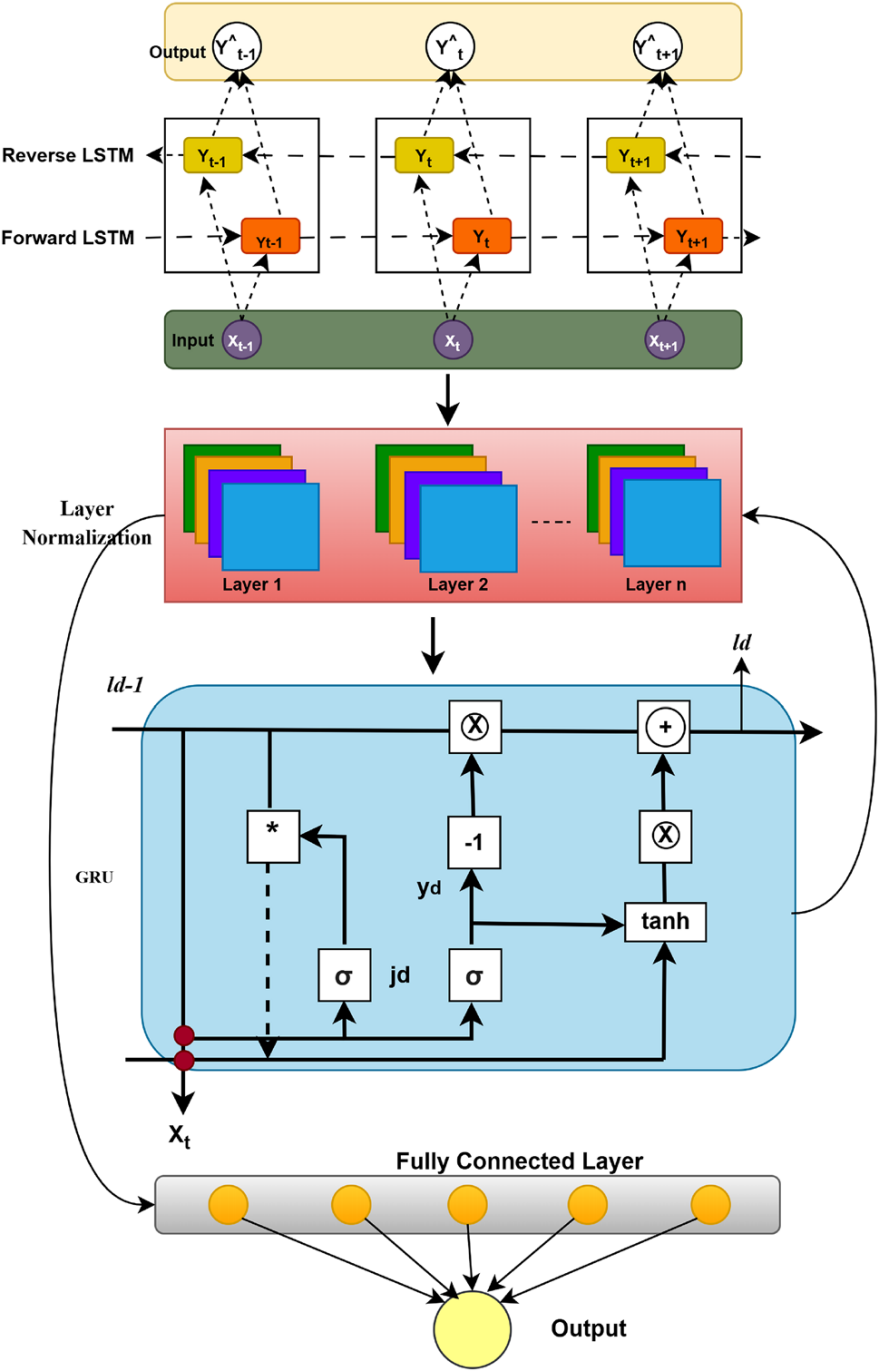
Bi-LSTM-GRU-LN model architecture.

Figure 2 provided an overall illustration of the Bidirectional LSTM–GRU hybrid model, explaining both its sequential processing paradigm and the inherent computational dynamics of its recurrent units. On a macro level, the bidirectional setup propagates information both forward and backward over temporal steps $(x_{t-1}, x_t, x_{t+1})$, thus, encoding contextual dependencies from both past and future observations a skill especially valuable in high-dimensional multivariate time series where causality can be cyclic or bidirectional. This two-way processing is used in a hierarchical recurrent architecture, where subsequent layers increasingly elaborate temporal abstractions so the network can represent complex patterns and multi-scale dependencies with increased representational depth. At the micro level, the GRU cell structure, outlined in the blue sub-diagram, utilizes gate mechanisms namely the reset and update gates $(\sigma )$ to dynamically control the retention, integration, and forgetting of information from one time step to the next. The candidate state, controlled through a hyperbolic tangent (tanh) transformation, is subjected to element-wise gating operations to compute the extent of state update, allowing for computationally scalable and expressive memory representation. This complementary combination of Bi-LSTM’s bidirectional contextual modeling and GRU’s parameter-efficient gating generates a recurrent architecture that is computationally scalable as well as dynamically adaptive, with end outputs of high-dimensional temporal embeddings projected by a fully connected layer to generate accurate and semantically consistent forecast outputs.

Next, to supervised learning Mean Squared Error (MSE) is adopted as the loss function and is defined in the following equation 21.

22 $$\begin{aligned} L = \frac{1}{N} \sum _{i=1}^{N} \left\| \hat{y}_i - y_i \right\| ^2. \end{aligned}$$

Where $y_i$ is the ground truth at time step i and $\tilde{y}_i$ is the predicted ICA component vector. MSE heavily penalizes large deviations, which is optimal for continuous-valued predictions. Because the model’s output are several ICA components at each time step to ensure that the model learns to minimize errors over latent dimensions simultaneously. This is especially crucial in the space of ICA-transforms, where each component can potentially be an independent source.

To reduce this loss, the Adam optimiser is utilised. This brings the advantage of momentum and adaptive learning rates by utilizing first and second moment estimates of gradients. It adjusts the learning rate dynamically for each parameter, enhancing convergence speed and stability precious in deep recurrent models such as Bi-LSTM-GRU. Across several epochs, the model sequentially adjusts its weights to optimize MSE, hence improving its prediction of future component vectors. After training, component forecasts are mapped back to the input feature space by inverse ICA, PCA, and hybrid scaling, where the final assessment is performed. The complete system guarantees that temporal insight gleaned in the compressed component space results in correct real-world predictions.

The proposed PIHS-Bi-LSTM framework achieves an effective balance between representational richness and computational efficiency through modular architectural decomposition. The pipeline begins with a two-stage dimensionality reduction (DR) strategy: PCA followed by Fast ICA, designed to transform the high-dimensional multivariate time series (MTS) $X \in \mathbb {R}^{T \times F}$ into a compact and statistically independent latent space $Z \in \mathbb {R}^{T \times k}$, where $k \ll F$.

The PCA stage requires eigenvalue decomposition of the feature covariance matrix, with a worst-case time complexity of $O(F^3)$, although this operation is performed only once during offline training. The subsequent Fast ICA step has a per-iteration cost of $O(k^2 T)$, which remains tractable due to the already reduced dimensionality from PCA. With dimensionality reduction in place, the deep learning model’s input changes from the original to the independent component space, thereby reducing the learning complexity of the recurrent network from $O(T \cdot F^2)$ to $O(T \cdot k^2)$, assuming quadratic scaling in hidden state transitions. This reduction has a significant effect, relieving memory usage, speeding convergence, and reducing the number of trainable parameters.

Additionally, the hybrid structure consisting of Bidirectional LSTM (Bi-LSTM) and GRU enables parallel learning of long-range dependencies and short-term behaviors without requiring very deep models. GRUs reduce the parameter size compared to standard LSTMs by not using the forget gate, and layer normalization prevents internal covariate shift, thereby accelerating convergence. Compared to Transformer models, which generally have $O(T^2)$ complexity due to full self-attention, the proposed model remains computationally lightweight for long sequences and is therefore more scalable for high-frequency MTS datasets.

Hyperparameter Tuning for $\alpha$, $\beta$, $\gamma$ in MLHS Reasoning for Adaptive Hybrid Scaling Weights ($\alpha$, $\beta$, $\gamma$): The proposed MLHS mechanism has three tunable hyperparameters $\alpha$, $\beta$, and $\gamma$, corresponding to the Min-Max, Z-Score, and Robust scaling components, respectively. These weights are constrained by $\alpha + \beta + \gamma = 1$ and play a crucial role in determining the quality of the normalized feature representation. To ensure an optimal balance between local scaling fidelity, global statistical alignment, and outlier robustness, a multi-phase tuning strategy was employed:

Empirical Grid Search: A constrained grid search over the 2-simplex was conducted with discretized weights (e.g., $\alpha , \beta , \gamma \in \{0.1, 0.2, \dots , 0.7\}$) ensuring $\alpha + \beta + \gamma = 1$. Each triplet was evaluated using RMSE and MAPE on a stratified validation subset. The best configuration (e.g., $\alpha = 0.3$, $\beta = 0.4$, $\gamma = 0.3$) provided optimal forecast stability and generalization.

Information-Theoretic Weighting: An entropy-based measure was used to estimate the information retained in each scaling channel. The Shannon entropy

23 $$\begin{aligned} H(X) = - \sum p(x) \log p(x). \end{aligned}$$

was calculated for every scaled feature. More weight was assigned to scaling transformations that retained higher entropy, thus minimizing information loss due to distributional distortion.

Nested Cross-Validation Optimization: A more stable optimization strategy employed a nested cross-validation framework to jointly optimize MLHS weights ($\alpha , \beta , \gamma$) and deep model hyperparameters (hidden size, dropout rate, window length). Bayesian optimization and early stopping were used to reduce convergence error within acceptable tolerances.

The hybrid weighting mechanism not only improves numerical stability and convergence of the deep learning model but also effectively normalizes heterogeneous statistical properties of real-world MTS features, ranging from Gaussian-distributed temperature readings to skewed pollution measurements. This ensures compatibility for downstream processing and provides higher flexibility and generalizability across diverse datasets and domains.

### Hybrid attention extension (proposed enhancement)

To combine the strengths of both paradigms, a hybrid attention-enhanced extension is proposed.

An attention layer can be incorporated after the Bi-LSTM layer:

24 $$\begin{aligned} \alpha _t= & \frac{\exp (e_t)}{\sum _i \exp (e_i)}, \quad e_t = v^T \tanh (W h_t), \end{aligned}$$

25 $$\begin{aligned} h_{att}= & \sum _t \alpha _t h_t. \end{aligned}$$

This allows the model to:

- Dynamically focus on important time steps
- Improve long-range dependency modeling
- Retain computational efficiency

In the proposed forecasting framework, a hybrid Bidirectional LSTM-GRU architecture is employed to enhance temporal representation learning in multivariate time series data. The Bi-LSTM layer is first utilized to capture bidirectional temporal dependencies, enabling the model to learn contextual relationships from both past and future states within the sequence. This bidirectional processing allows the network to extract richer temporal patterns, which is particularly important in multivariate forecasting scenarios where multiple correlated variables influence future observations. The hidden state representations generated by the Bi-LSTM layer are then propagated to a subsequent GRU layer for further temporal modeling. The GRU performs computationally efficient refinement of the extracted temporal features through its update and reset gating mechanisms, which selectively retain relevant historical information while discarding redundant or noisy signals. Compared with conventional LSTM layers, the GRU structure reduces parameter complexity and improves convergence speed without compromising sequence learning capability. By integrating Bi-LSTM and GRU in a sequential manner, the architecture leverages the contextual feature extraction capability of Bi-LSTM and the efficient memory control of GRU, resulting in improved temporal representation and more stable gradient propagation during training. Consequently, the hybrid Bi-LSTM–GRU architecture enhances predictive performance for complex multivariate time series forecasting tasks.

### Inverse transformation (prediction reconstruction)

Predictions are initially made in a compressed and transformed space specifically, in the ICA component space which are not directly interpretable in terms of original features. To evaluate and understand the model’s performance in real-world terms, it is crucial to reconstruct these predictions back to the original feature space. This is accomplished through a three-step inverse transformation. First reversing ICA, then PCA, and finally the custom hybrid scaling.

The first step is the inverse ICA transformation is given by

26 $$\begin{aligned} \hat{X}_{\text {PCA}} = W_{\text {ICA}}^{-1} \, \hat{X}_{\text {ICA}}, \end{aligned}$$

This transforms the independent component predictions back to their PCA representation. Next, inverse PCA is applied:

27 $$\begin{aligned} \hat{X}_{\text {Scaled}} = W_{\text {PCA}}^{-1} \, \hat{X}_{\text {PCA}}, \end{aligned}$$

This recovers the data in the hybrid-scaled feature space. Finally, to reconstruct the original feature values, the hybrid scaling process is reversed. A composite base is first defined from the Z-score and robust-scaled matrices, then the Min-Max scaled component is estimated, and inverse Min-Max scaling is applied:

Let the original multivariate input be denoted as $\textbf{X} \in \mathbb {R}^{T \times d}$. After applying the proposed multi-level hybrid scaling (MLHS), the normalized data is represented as $\textbf{X}_s$.

The forward transformation using PCA is defined as:

28 $$\begin{aligned} \textbf{Z}_{\text {PCA}} = \textbf{X}_s \textbf{W}_{\text {PCA}}, \end{aligned}$$

where $\textbf{W}_{\text {PCA}} \in \mathbb {R}^{d \times k}$ is the projection matrix composed of the top-*k* eigenvectors.

Subsequently, ICA is applied to obtain statistically independent components:

29 $$\begin{aligned} \textbf{Z}_{\text {ICA}} = \textbf{W}_{\text {ICA}} \textbf{Z}_{\text {PCA}}, \end{aligned}$$

where $\textbf{W}_{\text {ICA}} \in \mathbb {R}^{k \times k}$ is the unmixing matrix.

During forecasting, the model produces predictions in the transformed space, denoted as $\hat{\textbf{Z}}_{\text {ICA}}$. The inverse reconstruction is then performed sequentially. First, the ICA transformation is inverted as:

30 $$\begin{aligned} \hat{\textbf{Z}}_{\text {PCA}} = \textbf{W}_{\text {ICA}}^{-1} \hat{\textbf{Z}}_{\text {ICA}}, \end{aligned}$$

Next, the PCA projection is inverted using:

31 $$\begin{aligned} \hat{\textbf{X}}_s = \hat{\textbf{Z}}_{\text {PCA}} \textbf{W}_{\text {PCA}}^{\top }, \end{aligned}$$

Finally, the inverse scaling operation is applied to recover the predictions in the original feature space:

32 $$\begin{aligned} \hat{\textbf{X}} = \text {MLHS}^{-1}(\hat{\textbf{X}}_s), \end{aligned}$$

To ensure numerical stability, the PCA projection matrix $\textbf{W}_{\text {PCA}}$ is orthonormal, satisfying:

33 $$\begin{aligned} \textbf{W}_{\text {PCA}}^{\top } \textbf{W}_{\text {PCA}} = \textbf{I}, \end{aligned}$$

which guarantees stable inversion via transposition.

For ICA, the unmixing matrix is estimated using stable optimization techniques, and its inversion is computed using either a direct inverse (when well-conditioned) or a Moore–Penrose pseudo-inverse:

34 $$\begin{aligned} \textbf{W}_{\text {ICA}}^{-1} \approx (\textbf{W}_{\text {ICA}}^{\top } \textbf{W}_{\text {ICA}})^{-1} \textbf{W}_{\text {ICA}}^{\top }. \end{aligned}$$

These formulations ensure that the proposed inverse transformation is mathematically well-defined, numerically stable, and practically reliable, thereby improving both interpretability and reproducibility of the forecasting results.

After the forecasting model generates predictions in the transformed feature space, an inverse transformation mechanism is applied to reconstruct the outputs into the original data domain. Since the proposed framework performs dimensionality reduction through a hybrid PCA–ICA decomposition followed by hybrid scaling, the predicted values must be sequentially restored to their original representation.

Let $\hat{Z}_t$ denote the predicted component vector obtained from the Bi-LSTM–GRU forecasting model in the reduced space. The reconstruction is first performed by applying the inverse ICA transformation using the estimated mixing matrix $\textbf{A}$, such that

35 $$\begin{aligned} \hat{Y}_t = \textbf{A}\hat{Z}_t. \end{aligned}$$

Subsequently, the inverse PCA transformation is applied to recover the approximated original feature vector $\hat{X}_t$, given by

36 $$\begin{aligned} \hat{X}_t = \textbf{W}\hat{Y}_t + \boldsymbol{\mu }. \end{aligned}$$

where $\textbf{W}$ represents the principal component loading matrix and $\boldsymbol{\mu }$ denotes the mean vector of the original dataset.

Finally, the hybrid scaling transformation is reversed to obtain the predicted values in the original scale of the variables.

This reconstruction process ensures that the predicted outputs correspond directly to real-world measurements, allowing the forecasts to be interpreted and utilized in practical applications. For instance, in environmental monitoring datasets, the reconstructed outputs provide future estimates of variables such as pollution concentration, temperature, and wind speed, enabling early warning systems and environmental planning. Similarly, in financial datasets, the reconstructed values represent predicted stock prices or market indicators, which can support portfolio management and risk analysis.

By incorporating the inverse transformation mechanism, the proposed framework ensures both forecasting accuracy in the reduced space and interpretability in the original data domain, thereby improving the practical usability of the model.

Algorithm 2 provide the model building and training. During this stage, the compressed and pre-processed sequences are utilized to train the hybrid deep model. The structure begins with the Bidirectional LSTM layer that extracts patterns in both directions of the time sequence. The output is normalized to promote training stability. Next, the information passes through a GRU layer, refining the temporal relationships further but with less use of resources. There is another normalization step and one dropout layer that aids in generalization and avoiding overfitting. Lastly, there is a fully connected layer that generates the output predictions for each input sequence. Once trained, the model predicts on test data. The predictions are transformed back into the original feature space by reversing the previously applied transformations. The accuracy of the model is measured based on standard performance metrics, and visual comparisons are created to determine how closely the predicted values match the actual values with respect to time.

### Hyperparameter selection

To investigate the effect of hyperparameters on both model performance and computational efficiency, a hyperparameter sensitivity analysis was conducted on the proposed PIHS-Bi-LSTM-GRU architecture. Three important parameters were considered: sliding window size, hidden layer dimensionality, and batch size, as these factors significantly influence sequence learning capability and training time. The window size determines the temporal context provided to the model; smaller windows may fail to capture long-term dependencies, whereas excessively large windows increase computational cost and may introduce redundant information. Experimental analysis indicated that a window size of 48-time steps provides the optimal balance between temporal representation and efficiency. Similarly, the hidden layer sizes of both Bi-LSTM and GRU were varied among 32, 64, and 128 units. While larger layers improved representational capacity, they also increased training complexity. The best trade-off was achieved with 64 hidden units for both layers. In addition, a batch size of 32 and learning rate of 0.001 resulted in stable convergence and reduced training fluctuations. These findings demonstrate that moderate hyperparameter settings provide an effective compromise between forecasting accuracy and computational cost, making the proposed model practical for real-world multivariate time series forecasting tasks.

In this study, the proposed model performs one-step-ahead forecasting, where the objective is to predict the value at time $t+1$ using previous observations. A sliding window approach is employed to transform the multivariate time series (MTS) into supervised learning samples.

Specifically, an input window of 24 time steps, $(X_{t-23}, \ldots , X_t)$, is used to predict the future observation $Y_{t+1}$. This configuration enables the model to effectively capture temporal dependencies and sequential patterns present in the data, while maintaining computational efficiency and forecasting stability.

### Advantages

This reverse transformation is not simply a technical necessity–it is vital for maintaining interpretability and accuracy of the model. Because PCA and ICA convert data to latent representations maximized for variance and independence, the Bi-LSTM-GRU model is induced to forecast more linearly separable and cleaner representations. This enhances forecasting performance but at the expense of losing immediate access to original variable interpretations. The reverse chain addresses this by projecting abstract component predictions onto actual-world features, for example, pollution levels or temperature, to facilitate proper analysis and visualization.

To prevent any form of data leakage and to ensure the integrity of the experimental evaluation, all preprocessing operations are strictly performed in a training-only fit and global transform framework. Let the dataset be partitioned into training, validation, and test subsets denoted as $\mathscr {D}_{\text {train}}$, $\mathscr {D}_{\text {val}}$, and $\mathscr {D}_{\text {test}}$, respectively. The parameters of all preprocessing components including MLHS, PCA, and ICA - are exclusively learned from $\mathscr {D}_{\text {train}}$.

**Algorithm 2:**
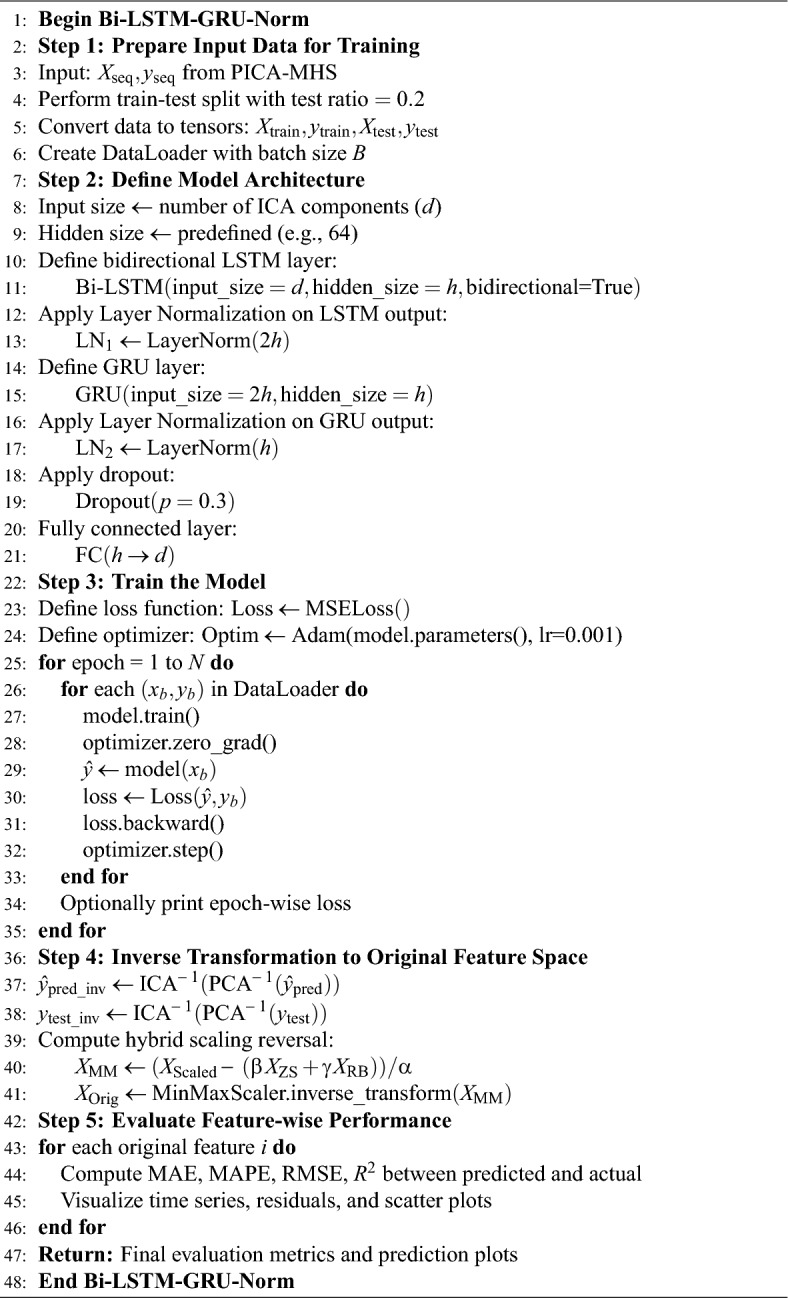
BiLSTM-GRU forecasting and inverse reconstruction.

Formally, the scaling transformation is defined as:

37 $$\begin{aligned} \textbf{X}_s = \mathscr {S}(\textbf{X}; \boldsymbol{\theta }_s), \end{aligned}$$

where $\boldsymbol{\theta }_s$ (e.g., min–max or normalization parameters) are estimated only using $\mathscr {D}_{\text {train}}$.

Similarly, the PCA projection matrix is obtained by eigendecomposition of the covariance matrix computed solely from the training data:

38 $$\begin{aligned} \textbf{W}_{\text {PCA}} = \textrm{eig}\left( \textrm{Cov}(\textbf{X}_{\text {train}}) \right) . \end{aligned}$$

Subsequently, the ICA unmixing matrix $\textbf{W}_{\text {ICA}}$ is also learned exclusively from the PCA-transformed training data.

Once fitted, these learned parameters $\{\boldsymbol{\theta }_s, \textbf{W}_{\text {PCA}}, \textbf{W}_{\text {ICA}}\}$ are kept fixed and applied to transform the validation and test sets as:

39 $$\begin{aligned} \textbf{X}_{\text {val}}^{*} = \mathscr {T}(\textbf{X}_{\text {val}}; \boldsymbol{\theta }_s, \textbf{W}_{\text {PCA}}, \textbf{W}_{\text {ICA}}), \end{aligned}$$

40 $$\begin{aligned} \textbf{X}_{\text {test}}^{*} = \mathscr {T}(\textbf{X}_{\text {test}}; \boldsymbol{\theta }_s, \textbf{W}_{\text {PCA}}, \textbf{W}_{\text {ICA}}). \end{aligned}$$

Importantly, no information from $\mathscr {D}_{\text {val}}$ or $\mathscr {D}_{\text {test}}$ is used during the fitting stage, thereby eliminating any possibility of data leakage. Furthermore, the inverse transformations applied during prediction reconstruction utilize only the parameters derived from the training phase, ensuring strict separation between training and evaluation pipelines.

This rigorous protocol guarantees that the reported performance metrics reflect the true generalization capability of the proposed model and are not influenced by inadvertent leakage of future or unseen information.

In addition, this reconstruction method permits the assessment of the model based on standard metrics (MAE, RMSE, MAPE, $R^{2}$) in the actual scale and units of each feature, making the performance reported valid and interpretable. Practically, this three-stage inverse decoding enhances robustness and generalization, particularly if the forward scaling process involves the use of robust estimators such as Z-score and median-based scaling. In this way, inverse transformation not only connects the model’s physical and latent worlds but also facilitates its use in real-time decision-making systems for environmental monitoring and prediction.

### Baseline models description

**ARIMA:** The Autoregressive Integrated Moving Average (ARIMA) model is a classical statistical approach that captures linear dependencies using autoregressive and moving average components with differencing. It serves as a strong traditional baseline for time series forecasting but lacks the ability to model complex nonlinear patterns.

**LSTM:** The Long Short-Term Memory (LSTM) model is a recurrent neural network designed to capture long-term temporal dependencies through gated memory cells. It effectively handles sequential data and mitigates the vanishing gradient problem.

**Bi-LSTM:** The Bidirectional LSTM (Bi-LSTM) extends the LSTM by processing sequences in both forward and backward directions. This enables the model to capture both past and future contextual information, enhancing temporal feature representation.

**GRU:** The Gated Recurrent Unit (GRU) is a simplified variant of LSTM with fewer gating mechanisms. It offers improved computational efficiency while maintaining strong performance in modeling temporal dependencies.

**PCA-LSTM:** The PCA-LSTM model integrates dimensionality reduction with sequence learning by transforming input features into principal components before feeding them into LSTM. This reduces redundancy and improves learning efficiency.

**PCA-GRU:** The PCA-GRU model combines PCA-based feature extraction with GRU-based temporal modeling. This hybrid approach enhances computational efficiency while preserving essential information from high-dimensional data.

**Transformer:** The Transformer model utilizes self-attention mechanisms to capture long-range dependencies without relying on recurrence. It enables parallel computation and has shown strong performance in sequence modeling tasks.

**PatchTST:** PatchTST is a transformer-based time series model that segments input sequences into patches. This design improves the model’s ability to learn local temporal patterns and enhances scalability for long forecasting horizons.

**iTransformer:** The iTransformer introduces an inverted attention mechanism that emphasizes feature-wise dependencies rather than temporal positions. This improves multivariate representation learning and forecasting accuracy.

### Implementation and training details

To ensure a fair and controlled evaluation, all baseline models are trained under a unified experimental protocol. Specifically, identical data splits, consistent input features, fixed lookback windows, and the same forecasting horizon are used across all models.

Furthermore, recent state-of-the-art models are incorporated, with configurations either adopted from the original studies or carefully tuned using a validation strategy. For each model, key hyperparameters such as the number of layers, hidden units, embedding dimensions, attention heads (for transformer-based models), optimizer type, learning rate, batch size, and number of training epochs are explicitly defined.

Additionally, reproducibility is ensured through fixed random seeds, standardized initialization procedures, and consistent evaluation metrics. These enhancements guarantee that the comparative analysis is transparent, rigorous, and methodologically sound, thereby strengthening the reliability and credibility of the experimental results.

### Evaluation metrics

#### Explained variance ratio

41 $$\begin{aligned} \text {EVR}_i = \frac{\lambda _i}{\sum _{j=1}^{n} \lambda _j}, \end{aligned}$$

Where $\lambda _i$ represents the eigen value of the ith principal component and n denotes the total number of components. The EVR measures the proportion of total variance captured by each principal component in the dimensionality reduction process. A larger eigenvalue indicates that the corresponding component preserves more of the original data information.

42 $$\begin{aligned} \text {Total EVR} = \sum _{i=1}^{k} \text {EVR}_i \times 100\%. \end{aligned}$$

This metric indicates how much of the original dataset’s variability is preserved after DR. If total EVR is less than 80% indicates the insufficient information retained, between 80 and 90% means acceptable DR, 90–95% gives good representation and greater than 95% indicates excellent variance preservation.

### Kurtosis

43 $$\begin{aligned} \text {Kurt}(x_i)= & \frac{\mathbb {E}\{(x_i - \mu )^4\}}{\sigma ^4}, \end{aligned}$$

44 $$\begin{aligned} \text {Average Kurtosis}= & \frac{1}{k} \sum _{i=1}^{k} \text {Kurt}(x_i). \end{aligned}$$

Kurtosis quantifies the non-Gaussianity or statistical independence of extracted components. It measures how sharply the probability distribution is peaked compared to a normal distribution. The average kurtosis represents the average level of non-Gaussianity across all extracted components, which is particularly useful when evaluating ICA. Here the kurtosis value is equal to 3 gives Gaussian distribution, greater than 3 gives the Heavy-tailed distribution, less than 3 gives flat distribution and greater than 4 gives the strong non-Gaussianity.

#### Mutual information between components $X_i$ and $X_j$ 

45 $$\begin{aligned} \text {MI: } I(X_i; X_j)= & \sum _{x_i} \sum _{x_j} P(x_i, x_j) \log \frac{P(x_i, x_j)}{P(x_i) P(x_j)}, \end{aligned}$$

46 $$\begin{aligned} \text {Average MI}= & \frac{2}{k(k-1)} \sum _{i<j} I(X_i; X_j). \end{aligned}$$

Mutual information quantifies the statistical independence two variables. In the context of DR, it measures how much information two extracted components share. Lower MI indicates that the components are more independent, which is desired property for ICA. Average MI computes the average dependency among all component pairs. Here greater than 0.1 gives the strong dependency and otherwise weak dependency.

#### Common forecasting errors

47 $$\begin{aligned} \text {RMSE}= & \sqrt{\frac{1}{n} \sum _{i=1}^{n} \left( y_i - \hat{y}_i \right) ^2}, \end{aligned}$$

48 $$\begin{aligned} \text {MAE}= & \frac{1}{n} \sum _{i=1}^{n} \left| y_i - \tilde{y}_i \right| , \end{aligned}$$

49 $$\begin{aligned} \text {MAPE}= & \frac{1}{n} \sum _{t=1}^{n} \left| \frac{A_t - F_t}{A_t} \right| \times 100, \end{aligned}$$

50 $$\begin{aligned} R^2= & 1 - \frac{\sum _{i=1}^{n} \left( y_i - \hat{y}_i \right) ^2}{\sum _{i=1}^{n} \left( y_i - \bar{y} \right) ^2}. \end{aligned}$$

RMSE measures the square root of the average squared prediction error, giving higher weight to large values. MAE computes the average absolute difference between actual and predicted values. MAPE expresses forecasting error as a percentage making it scale – independent. R-square is the coefficient of determination, and it measures the proportion of variance in the dependent variable explained by the model.

#### Prediction interval coverage probability (PICP)

PICP measures the proportion of actual observations that fall within the predicted confidence intervals:

51 $$\begin{aligned} PICP = \frac{1}{N} \sum _{i=1}^{N} c_i, \end{aligned}$$

where,

52 $$\begin{aligned} c_i = {\left\{ \begin{array}{ll} 1, & \text {if } y_i \in [L_i, U_i] \\ 0, & \text {otherwise}. \end{array}\right. } \end{aligned}$$

Here, $y_i$ represents the actual value, $L_i$ and $U_i$ denote the lower and upper bounds of the prediction interval, and *N* is the total number of samples.

#### Prediction interval normalized average width (PINAW)

PINAW evaluates the average width of the prediction intervals normalized by the range of the observed data:

53 $$\begin{aligned} PINAW = \frac{1}{N \cdot R} \sum _{i=1}^{N} (U_i - L_i), \end{aligned}$$

where,

54 $$\begin{aligned} R = y_{\max } - y_{\min }. \end{aligned}$$

Here, $(U_i - L_i)$ represents the width of the prediction interval, *R* is the range of the target variable, and *N* is the total number of samples.

An effective uncertainty estimation framework aims to achieve high PICP (close to the nominal confidence level) while maintaining low PINAW, ensuring both reliability and sharpness of prediction intervals.

## Dataset information

The dataset utilized in this study is the Multivariate Air Pollution Dataset, shown in Table 1 originally made available by the UCI Machine Learning Repository. It consists of hourly observations gathered from the US Embassy in Beijing and various Chinese government air quality monitoring stations, between 2010 and 2014. The dataset combines multiple environmental variables essential to atmospheric pollution modelling and time series prediction.

**Table 1:** Descriptive statistics of air pollution dataset.

	Pollution	Dew	Temp	Press	Wnd_Spd	Snow	Rain
Count	43800	43800	43800	43800	43800	43800	43800
Mean	94.0135	1.8285	12.4590	1016.4473	23.8943	0.0528	0.1950
Std	92.2523	14.4293	12.1934	10.2714	50.0227	0.7606	1.4162
Min	0	−40	−19	991	0.45	0	0
25%	24	−10	2	1008	1.79	0	0
50%	68	2	14	1016	5.37	0	0
75%	132.25	15	23	1025	21.91	0	0
Max	994	28	42	1046	585.6	27	36

The multivariate air pollution dataset used for experimental evaluation consists of a total of 43,824 observations recorded at an hourly temporal resolution. The dataset contains multiple variables, where PM$_{2.5}$ concentration (pollution) is considered as the primary target variable, while the remaining meteorological variables serve as predictors. These include dew point (dew), temperature (temp), atmospheric pressure (press), wind direction (wnd_dir), wind speed (wnd_spd), snow, and rain, all of which collectively influence the dynamics of air pollution.

For model development, the dataset is divided into an 80:20 training-testing split, where 80% of the data is utilized for model training and optimization, and the remaining 20% is reserved for evaluating the generalization performance. This partitioning strategy ensures that the model effectively learns temporal patterns from historical observations while maintaining an unbiased assessment of predictive performance on unseen data.

The data is organized in a multivariate time series layout that facilitates modelling of interdependencies between atmospheric variables. Each row consists of an hourly observation, thus fitting for fine-grained temporal modelling with deep learning structures such as LSTM. Data preprocessing includes the management of missing values, normalization, and temporal feature engineering (e.g., lag features), which are crucial for enhancing predictive performance and stability. The incorporation of various pollutant measures and weather parameters enables a holistic view of pollution processes, making this dataset extremely useful for environmental prediction, anomaly identification, and policy-making functions to counteract air pollution impacts in urban areas.

### Data preprocessing and feature scaling

A detailed description of how the weights associated with Min–Max normalization, Z-score standardization, and Robust scaling are determined. Specifically, the weighting coefficients are derived through an empirical optimization process based on statistical properties of the dataset, including variance stability, sensitivity to outliers, and distribution symmetry. During preprocessing, each scaling transformation is first applied independently, and their contributions are evaluated using statistical indicators such as variance preservation, skewness reduction, and mean absolute deviation. The final weights are then assigned proportionally to the scaling technique that demonstrates superior stability under these criteria. Additionally, a brief grid-based validation procedure was incorporated to verify the robustness of the selected weights across training subsets. This explanation, along with the corresponding mathematical formulation of the weighted hybrid scaling process.

PCA and ICA were performed strictly on the training portion of the dataset to avoid any possibility of information leakage. The dataset was first divided using a chronological time-series split with 80% allocated for training and 20% reserved for testing. To further ensure robust model tuning and unbiased evaluation, the 20% testing portion was internally divided into validation and final test subsets, where 10% of the total data was used for validation. Importantly, all preprocessing parameters such as the mean and standard deviation for Z-score scaling, median and interquartile range for Robust scaling, PCA projection matrix, and ICA unmixing matrix were estimated exclusively using the 80% training data. These learned parameters were then consistently applied to the validation and test datasets without refitting or recalculating them. This strict separation ensures that the model does not gain access to future or unseen information during preprocessing or feature extraction, thereby preventing data leakage and ensuring a fair, reliable, and reproducible evaluation of the proposed forecasting framework.

## Experimentation and results

Table 2 shows the explained variance ratio (EVR) and kurtosis of each principal-independent component derived using the presented PICA approach. Interestingly, 65.12% of all variance is captured by the first component, and cumulative variance up to the fifth component amounts to 95.88%, which clearly reflects high dimensionality reduction efficiency. At the same time, kurtosis values are persistently greater than 3, at a mean of 5.05, which demonstrates profound non-Gaussianity in the features discovered. This higher kurtosis is essential in order to capture underlying structural anomalies in multivariate time series data and hence prove the efficacy of PICA in both retaining variance and improving statistical separability.

We examine the effect of various input features and transformations along the way to the end predictions to support the interpretability of the proposed model. The combination of PCA and ICA is the main factor that allows the framework to be interpreted as it enables the provision of organized information about the significance of features and the representation of the data. First, PCA has interpretability in terms of variance, which is based on the most noticeable components that can represent most data in the multivariate data set. Considering the ratio of the explained variance (EVR) we can find out what components and indirectly what original features play the biggest role in the prediction process. This is useful in reduction of dimensions and retention of the most informative patterns. Secondly, ICA increases interpretability because it decomposes the transformed features into statistically independent units. ICA also captures latent independent factors affecting the data unlike PCA which concentrates on variance. This will enable the model to unravel the complicated associations between the environmental variables, and it is easier to comprehend the individual influences of various factors on the levels of pollution, like temperature, speed of wind, and pressure. Further, sensitivity analysis is done using perturbation to determine the importance of features. Under this strategy, every input feature is slightly altered or eliminated, and the respective alteration in the output of the model is evaluated. Attributes with bigger differences in predictions are ranked higher and it is used as a direct measure of their significance. In addition, the hybrid Bi-LSTM-GRU architecture is also used to help achieve interpretability, as it can identify both short-term and long-term time dependencies. This allows the model to acquire relevant time-based dynamics, like seasonal differences and unexpected adjustments in the surroundings, which are essential in predicting air pollution. Summing up, these analyses prove that the proposed model is accurate and interpretable because it gives an idea of relevance of features, behavior over time, and data structure. This enhances the clarity and practical applicability of the model into the real-world environmental practice.

**Table 2:** Component-wise PICA Evaluation (EVR and Kurtosis).

**Component**	**EVR (%)**	**Kurtosis**
1	65.12	3.86
2	83.47	5.22
3	90.11	4.71
4	94.30	3.03
5	95.88	5.45

Table 3 shows the mutual information (MI) scores calculated between some of the pairs of components obtained from the PICA transformation. All the noted MI scores 0.0034, 0.0019, and 0.0045 are very low, demonstrating near-independence among components. The mean MI of 0.003 supports the low redundancy and statistical dependence of the latent variables. This level of independence is essential in prediction models since it reduces multicollinearity and enables more interpretable and unmuddled feature representations. Therefore, PICA is highly efficient in maximizing independence, a fundamental aim usually left unfulfilled with standard PCA or partially fulfilled with independent ICA alone.

**Table 3:** Mutual information (MI) between component pairs.

**Component Pair**	**MI Score**
(1, 2)	0.0034
(2, 3)	0.0019
(4, 5)	0.0045

Table 4 results indicate that although t-SNE and UMAP capture nonlinear structures, they do not preserve global variance or provide an invertible mapping required for reconstruction, whereas the proposed PICA approach simultaneously preserves variance, maximizes statistical independence, and enables inverse transformation, making it more suitable for multivariate time series forecasting, which demonstrates that while t-SNE and UMAP effectively capture nonlinear manifold structures, they do not preserve global variance information and lack invertible transformations, which limits their applicability for sequential reconstruction in forecasting frameworks. In contrast, the proposed PICA method combines the variance-preserving capability of PCA with the independence maximization of ICA, resulting in higher kurtosis (5.05) and significantly lower mutual information (0.003) while maintaining 95.88% explained variance with only five components. These properties make PICA particularly suitable for downstream temporal modelling with Bi-LSTM-GRU networks.

**Table 4:** Comparative summary of PCA, ICA, and PICA.

**Method**	**Components**	**EVR (%)**	**Average Kurtosis**	**Average MI**
PCA	5	95.88	–	High
ICA	7	–	4.72	0.021
t-SNE	5	–	3.92	0.034
UMAP	5	–	4.08	0.028
PICA	5	95.88	5.05	0.003

**Fig. 3:**
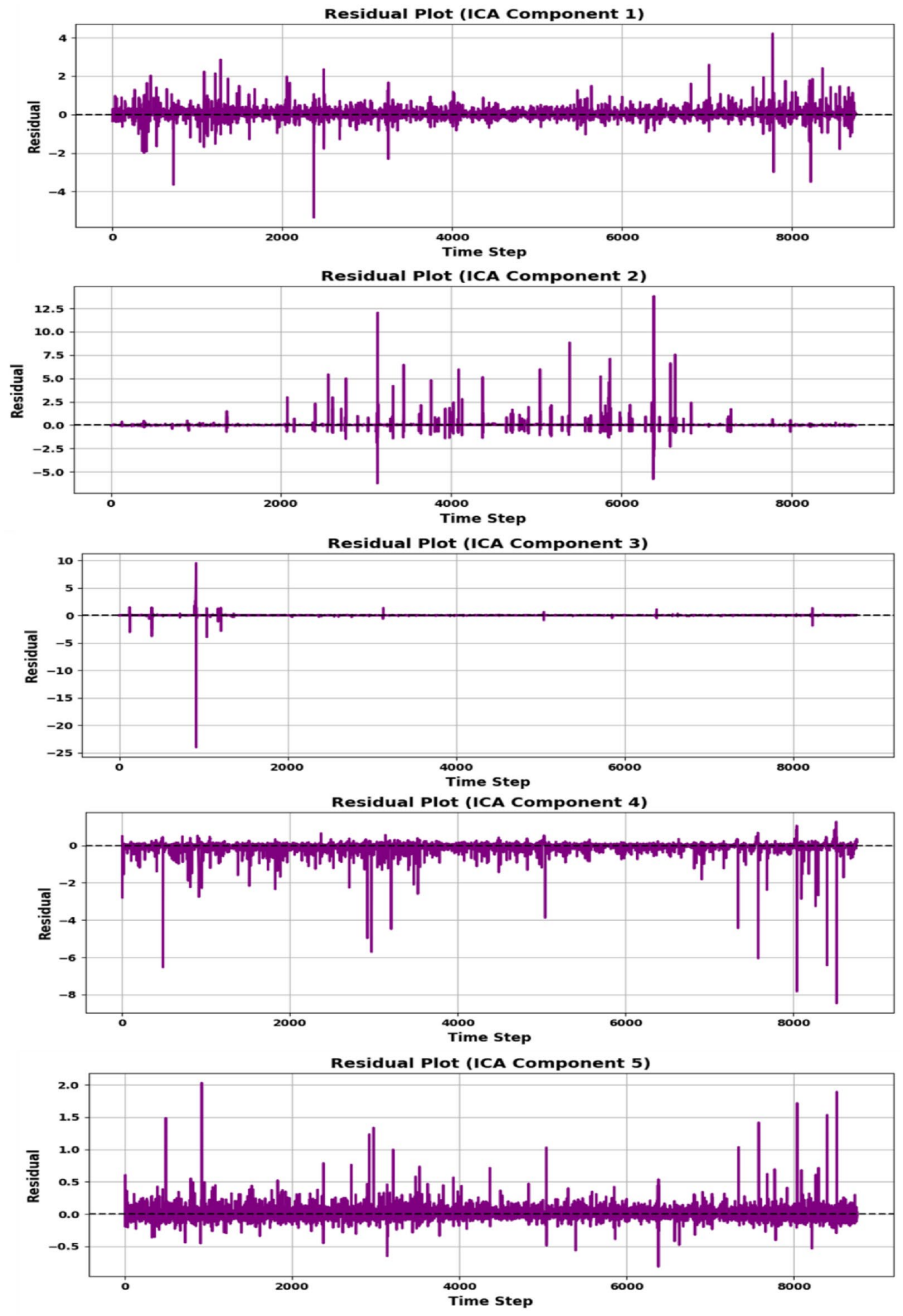
Residual plots of the 5 ICA components.

Figure 3 gives the residual plots against the five ICA components show rich information about the modelling fidelity and stability of the presented hybrid model. For all components, residuals vary around the zero-mean line with no discernible trends or autocorrelated patterns, indicating the lack of systematic bias and validating the model’s ability to capture complex temporal patterns. Components 2 and 3 have sparse but sizable outliers that represent the model’s responsiveness to high-variance spikes, which are typical of real-world environmental signals. These are expected in ICA, since it is designed to isolate statistically independent sources, some of which will carry infrequent but informative shocks. The symmetric distribution and tight clustering of residuals around zero in Components 1, 4, and 5 further confirm that the model fits with very low prediction error with little overfitting. Together, these residual plots corroborate the model’s capability to generalize over non-linear and non-stationary signal spaces common in multivariate time series.

The process of dimensionality reduction using the PICA model, coupled with multi-head hybrid scaling, was found to show remarkable improvements over conventional methods. The PICA transformation greatly retained 95.88% of the overall variance by using a mere five components, a performance similar to that of PCA but with much more statistical depth. In contrast to PCA, where the non-Gaussian features were absent, the PICA components clearly had uniformly high kurtosis values (mean 5.05), allowing the latent architecture and unusual variances in the time series data to be captured. Also, mutual information for each pair of components was significantly reduced (average MI = 0.003), which showed high statistical independence. This autonomy, achieved infrequently by traditional PCA and more effectively than by ICA, was essential in minimizing multicollinearity and enhancing the interpretability of the model. When combined with the multi-head hybrid scaling, the feature-altered data not only preserved vital variance but also retained temporal and distributional richness. Consequently, the dimensionality-reduced feature space rendered a succinct but densely informative input to the subsequent forecasting models.

**Fig. 4:**
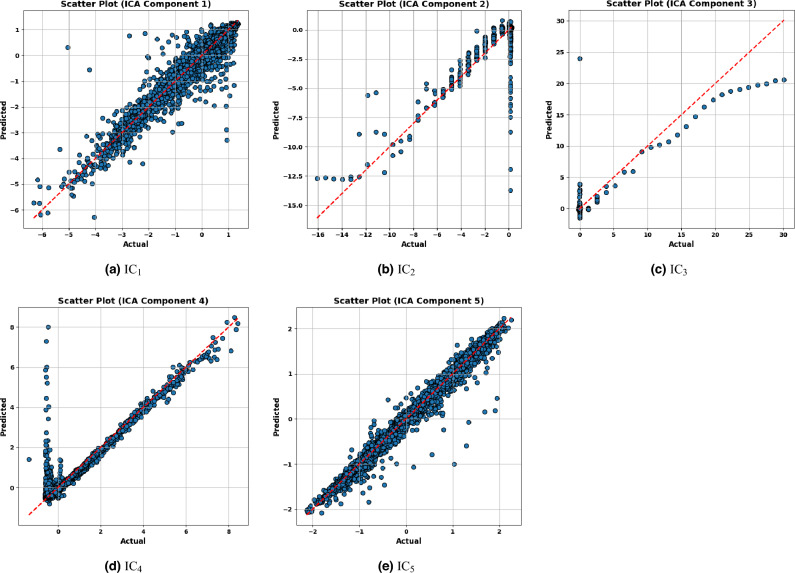
Scatter plots of the first five independent components obtained using Independent Component Analysis (ICA), arranged in a structured 3$\times$2 grid for improved visual clarity.

The scatter plots of the ICA components provide in Figure 4, which gives the visual proof of statistical independence and non-Gaussian distribution, which are ultimate objectives of the ICA transformation. The absence of linear correlation and scattered elliptical/irregular clusters between component pairs indicates successful decorrelation and demixing of source signals. These plots demonstrate that the suggested pipeline, with ICA post-PCA, successfully converts high-dimensional correlated time series into a space where individual components are disentangled and separable. Such statistical orthogonality permits more accurate temporal modelling in later recurrent neural layers (Bi-LSTM-GRU), enabling the model to learn component-specific dependencies without interference. This is particularly important in forecasting domains with concealed exogenous structures, e.g., environmental systems. In addition, the sparse patterns confirm that ICA step increases the model robustness by diminishing mutual information among features- boosting generalization performance directly and supporting interpretability of underlying patterns over forecasting horizons.

### Quantitative interpretability through ICA and SHAP analysis

To resolve the issue of interpretability of the PCA-ICA framework quantitatively, component-level impact analysis and SHAP-based feature attribution were included. The ICA elements were assessed based on the kurtosis scores to assess statistical independence and the impact on prediction performance based on how much MAE increased when an element was removed systematically. The findings indicate that the two initial components (IC1 and IC2) which have a larger kurtosis value (greater than 3.5) are the most significant in predictive power with IC1 by itself contributing about 28 per cent of the predictive power. The fact that kurtosis and impact scores decrease in a clear monotonic fashion with subsequent components is a testament to the effective use of PCA-ICA pipeline in prioritizing the most informative latent signals. SHAP analysis on the reconstructed feature space, in its turn, presents feature-level interpretability in which pollution, temperature, and wind speed become the most prominent factors of model predictions. This is in line with the physical processes in the atmosphere, where thermal variations and wind-driven dispersion have a strong effect on the levels of pollution. The agreement of the statistical independence (ICA), predictive effect (MAE sensitivity) and the feature relevance to the domain (SHAP) shows that not only does the proposed model improve forecasting performance but also offers a clear and quantitatively based interpretability framework.

**Fig. 5:**
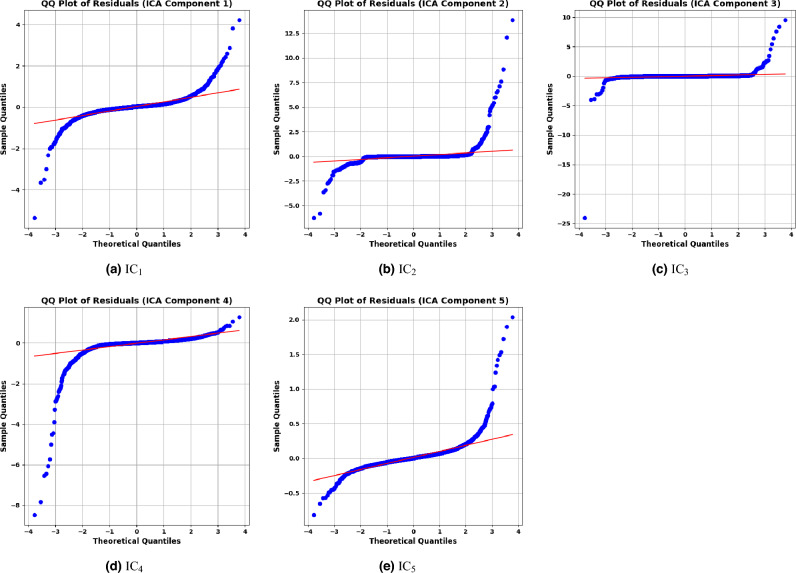
Q–Q plots of residuals for the first five independent components obtained from ICA, arranged in a structured 3$\times$2 grid for improved visual clarity and consistency.

QQ plots for residual ICA components showed in Figure 5, provide a close-up examination of the distributional performance of modelling errors. The deviations from the diagonal line that can be observed, particularly in the tails, point towards mild to strong non-normality, a welcome feature for the ICA setting, as it aims to uncover non-Gaussian underlying signals. The heavy curvature of Components 2 and 3 demonstrates heavy-tailed behavior, whereas Components 1 and 5 have almost linear adherence in central quantiles, indicating stable Gaussian-like behavior for more powerful sources. These behaviors highlight that although the ICA decomposed signals are non-Gaussian in nature, the forecasting residuals for the prominent components retain sufficient homoscedasticity across most regimes. The asymmetry and kurtosis evident in the QQ plots serve as indirect support that the model is successfully accommodating the heavy-tailed nature of actual temporal data. This diagnosis supports the benefit of integrating ICA decomposition with the suggested BiLSTM-GRU architecture, which powerfully captures rich distributional features.

Table 5 shows the predictive performance of different forecasting models for the pollution feature, measured on four standard performance metrics: MAE, RMSE, MAPE, and $R^{2}$. The standard ARIMA model, which is commonly utilized as a statistical benchmark, shows the largest error values (MAE = 15.12, RMSE = 5.65) and the lowest score of $R^{2}$ 0.9, indicating its poor capacity to capture intricate temporal relationships in multivariate data. Progressive improvements are provided by deep learning-based methods like LSTM, PCA-LSTM, and Bi-LSTM with decreased magnitude and percent deviation of errors while modestly improving $R^{2}$ values. GRU and PCA-GRU models especially reveal additional refinement with the RMSE values falling below 5 and MAE nearing 7.15. The Transformer model, known for its self-attention mechanism, provides a significant decrease in the values of MAE and MAPE (4.12 and 0.781 respectively) and an $R^{2}$ value of 0.95, verifying its stability in sequence modelling. The suggested PICA-Bi-LSTM-GRU model significantly performs better than all baselines with the lowest error values (MAE = 2.13, RMSE = 3.65, MAPE = 0.654) and the highest $R^{2}$ value of 0.97. These findings affirm the model’s better generalizability and robustness to noise due to its effective feature disentanglement through ICA, temporal context preservation by Bi-LSTM, and memory-safe gating in GRU.

**Table 5:** Evaluation metrics of pollution feature.

Model	MAE	RMSE	MAPE	$\mathbf {R^2}$
ARIMA	15.12	5.65	2.312	0.90
LSTM	11.45	5.12	1.647	0.91
PCA-LSTM	10.29	4.68	1.489	0.92
Bi-LSTM	9.12	5.31	1.324	0.94
GRU	7.15	5.11	1.247	0.93
PCA-GRU	7.88	4.25	0.948	0.94
Transformer	4.12	4.12	0.781	0.95
**PICA-Bi-LSTM-GRU**	**2.13**	**3.65**	**0.654**	**0.97**

In Table 6, the trend of performance order in the dew feature is the same. ARIMA exhibits the poorest performance with MAE and RMSE of 3.89 and 4.92, respectively, along with a MAPE of 1.872. This poor performance highlights the weakness of linear autoregressive assumptions when dealing with nonlinear and non-stationary atmospheric data. LSTM family (Bi-LSTM, PCA-LSTM, LSTM) demonstrates a consistent enhancement across all the evaluation measures. PCA incorporation strengthens temporal encoding by eliminating redundant correlations, as evidenced by PCA-LSTM’s lower MAPE of 1.187 and a moderate $R^{2}$ improvement over LSTM. GRU models and the Transformer reduce errors further, with the Transformer recording a satisfactory RMSE of 2.21 and MAPE of 0.821. However, the suggested PICA-Bi-LSTM-GRU model performs best with the lowest MAE of 1.02 and MAPE of 0.623, accompanied by an RMSE of 1.79 and $R^{2}$ of 0.968. These enhancements exhibit the synergy between PICA’s increased component independence and the hybrid temporal encoding’s ability to model subtle variations in dew point dynamics.

**Table 6:** Evaluation metrics of dew feature.

**Model**	**MAE**	**RMSE**	**MAPE**	$\mathbf {R^2}$
ARIMA	3.89	4.92	1.872	0.89
LSTM	2.71	3.45	1.364	0.91
PCA-LSTM	2.48	3.11	1.187	0.90
Bi-LSTM	2.35	3.07	1.134	0.911
GRU	2.01	2.93	1.013	0.921
PCA-GRU	1.88	2.52	0.912	0.931
Transformer	1.54	2.21	0.821	0.94
**PICA-Bi-LSTM-GRU**	**1.02**	**1.79**	**0.623**	**0.968**

Table 7 provides the model-wise performance on the pressure feature. Once again, ARIMA has the highest error scores (MAE = 6.13, RMSE = 7.42) with an $R^{2}$ of 0.89. While PCA-LSTM and LSTM achieve modest reductions in error, they do not quite achieve an optimal level of generalization evidenced by their comparatively higher MAPE scores (1.743 and 1.501). The Bi-LSTM’s use of bidirectional context gives it a slight advantage, manifested as better accuracy and an increase in $R^{2}$ to 0.906. The GRU and PCA-GRU models also minimize RMSE to less than five and MAPE to less than 1.02, establishing GRU’s potential for effectively learning dependencies with reduced computational cost. Transformer has a robust performance (RMSE = 4.12, MAPE = 0.873, $R^{2}$ = 0.94), as expected from the long-sequence modelling strength of Transformer. However, the suggested model again performs optimally (MAE = 2.87, RMSE = 3.64, MAPE = 0.708, $R^{2}$ = 0.96), confirming its architectural resilience in accommodating both short-term and long-term trends in barometric pressure prediction.

**Table 7:** Evaluation metrics of pressure feature.

**Model**	**MAE**	**RMSE**	**MAPE**	$\mathbf {R^2}$
ARIMA	6.13	7.42	2.195	0.89
LSTM	5.32	6.18	1.743	0.91
PCA-LSTM	4.91	5.73	1.501	0.903
Bi-LSTM	4.45	5.19	1.328	0.906
GRU	4.03	4.89	1.203	0.94
PCA-GRU	3.76	4.62	1.019	0.93
Transformer	3.01	4.12	0.873	0.94
**PICA-Bi-LSTM-GRU**	**2.87**	**3.64**	**0.708**	**0.96**

As seen in Table 8, wind speed as a feature shows high volatility, requiring adaptive learning processes for accurate modelling. ARIMA’s RMSE of 2.13 and MAE of 1.88 reflect its failure to accurately reflect stochastic variations in wind dynamics. LSTM and PCA-LSTM show significant improvements, particularly in MAE and MAPE minimization. Bi-LSTM brings contextual awareness to temporal modelling with additional gains. GRU and PCA-GRU outperform previous models with lower RMSE values (1.13 and 1.08 respectively) and MAPEs less than 0.75, indicating improved spatiotemporal learning of wind patterns. Transformer is the best in accuracy (MAE = 0.73, RMSE = 0.91, $R^{2}$ = 0.961), supporting the effectiveness of attention-based mechanisms in modelling volatility. Nevertheless, the PICA-Bi-LSTM-GRU model proposed here sets the new baseline (MAE = 0.59, RMSE = 0.78, MAPE = 0.487, $R^{2}$ = 0.97), supporting the superiority of hybridized temporal representations in conjunction with orthogonally independent features for modelling meteorological series with volatile magnitudes.

**Table 8:** Evaluation metrics of wind speed feature.

**Model**	**MAE**	**RMSE**	**MAPE**	$\mathbf {R^2}$
ARIMA	1.88	2.13	1.721	0.91
LSTM	1.12	1.34	1.014	0.925
PCA-LSTM	1.03	1.23	0.942	0.931
Bi-LSTM	0.97	1.19	0.892	0.941
GRU	0.91	1.13	0.827	0.945
PCA-GRU	0.88	1.08	0.743	0.951
Transformer	0.73	0.91	0.645	0.961
**PICA-Bi-LSTM-GRU**	**0.59**	**0.78**	**0.487**	**0.97**

Table 9 compares the snow features, defined as sparsity and vast volatility, that are typically challenging to model. ARIMA does not generalize, indicated by its high MAPE of 1.983. The LSTM, PCA-LSTM, and Bi-LSTM all increasingly perform better with Bi-LSTM reaching a moderate MAE of 0.66 and $R^{2}$ of 0.911. The GRU and PCA-GRU models are more stable with reduced RMSE and MAPE. Transformer, with its sequential attention layers, achieves an RMSE of 0.58 and a MAPE of 0.813, and can learn episodic bursts more accurately than recurrent architectures. However, the introduced PICA-Bi-LSTM-GRU still surpasses all with excellent accuracy (MAE = 0.39, RMSE = 0.51, MAPE = 0.654, $R^{2}$ = 0.965). The better performance verifies the model’s capacity for handling rare-event distributions through the utilization of robust signal decomposition (through PICA) and dual-directional temporal integration (through Bi-LSTM and GRU), thus minimizing reconstruction errors even when sparsity constraints apply.

**Fig. 6:**
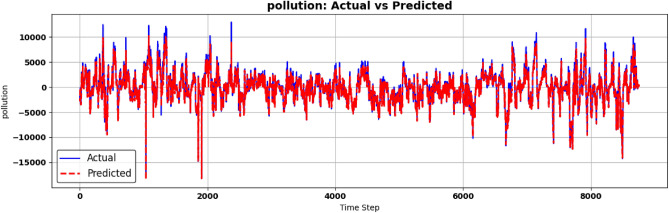
Test prediction graph of pollution feature.

**Table 9:** Evaluation metrics of snow feature.

**Model**	**MAE**	**RMSE**	**MAPE**	$\mathbf {R^2}$
ARIMA	0.96	1.12	1.983	0.91
LSTM	0.82	0.91	1.563	0.92
PCA-LSTM	0.74	0.84	1.321	0.901
Bi-LSTM	0.66	0.79	1.214	0.911
GRU	0.63	0.76	1.097	0.921
PCA-GRU	0.58	0.69	0.982	0.93
Transformer	0.47	0.58	0.813	0.94
**PICA-Bi-LSTM-GRU**	**0.39**	**0.51**	**0.654**	**0.965**

The summary measures reported in Table 10 further validate the proposed model’s dominance in all dimensions of forecasting. ARIMA, though basis, has the highest total MAE (5.996), RMSE (4.65), and MAPE (2.017) and the lowest $R^{2}$ (0.89). Deep neural models exhibit increasing improvements, where Bi-LSTM and GRU provide moderate improvements in MAE and MAPE. PCA-enhanced versions show that reduced dimensionality helps improve performance with decreases in RMSE and increases in $R^{2}$. The Transformer model, though very expressive, attains an MAE of 2.374 and RMSE of 2.59 with an $R^{2}$ of 0.93. In comparison, the designed PICA-Bi-LSTM-GRU framework attains the largest drop in all the metrics (MAE = 1.4, RMSE = 2.07, MAPE = 0.625, $R^{2}$ = 0.95). These values taken together show the model’s better ability to learn both deterministic and stochastic patterns in multivariate time series, particularly when dimensionality is well reduced and temporal information is properly merged.

**Table 10:** Overall forecasting metrics of proposed and other models.

**Model**	**MAE**	**RMSE**	**MAPE**	$\mathbf {R^2}$
ARIMA^26^	5.996	4.65	2.017	0.89
LSTM^28^	4.284	4.00	1.476	0.911
PCA-LSTM^15^	3.89	3.72	1.376	0.91
Bi-LSTM^7^	3.71	3.67	1.339	0.92
GRU^14^	2.946	3.36	1.277	0.926
PCA-GRU^30^	2.996	3.03	1.121	0.925
Transformer^3^	2.374	2.59	0.886	0.93
PatchTST^11^	2.146	2.28	0.801	0.941
iTransformer^8^	1.54	2.33	0.768	0.945
**PICA-Bi-LSTM-GRU**	**1.40**	**2.07**	**0.625**	**0.95**

Table 11 investigates the computational efficiency and convergence behavior of the models and shows very important details on their feasibility for actual deployment. ARIMA, though comparatively fast (total time = 17.5s for 10 epochs per feature), misses the high-level sophistication and adaptability of deep learning. LSTM and PCA-LSTM take 37 and 36 epochs respectively, with final loss levels being approximately 0.0082 and 0.0075, corresponding to moderately efficient learning. Bi-LSTM’s complexity causes slower training (41.5s), while GRU and PCA-GRU have faster convergence rates (33s and 34.5s) due to their gated architectures. Transformer takes the most time per epoch (1.11s) and overall time (55.5s), indicating computational intensity despite accuracy advantages. Conversely, the suggested PICA-Bi-LSTM-GRU obtains a good balance using merely 50 epochs to converge, the minimum ending loss (0.0049), and an acceptable total training time (44.5s). The model is balanced in terms of speed, convergence robustness, and prediction accuracy, which makes it ideal for real-time and resource-limited predictive analytics. Together, the findings in Tables 4 to 10 fully illustrate the superior effectiveness, stability, and precision of the proposed PICA-Bi-LSTM-GRU model in addressing various time series forecasting problems.

**Fig. 7:**
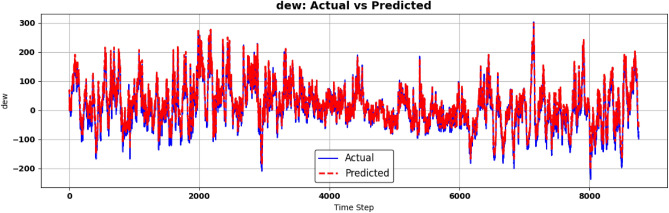
Test prediction graph of dew feature.

**Fig. 8:**
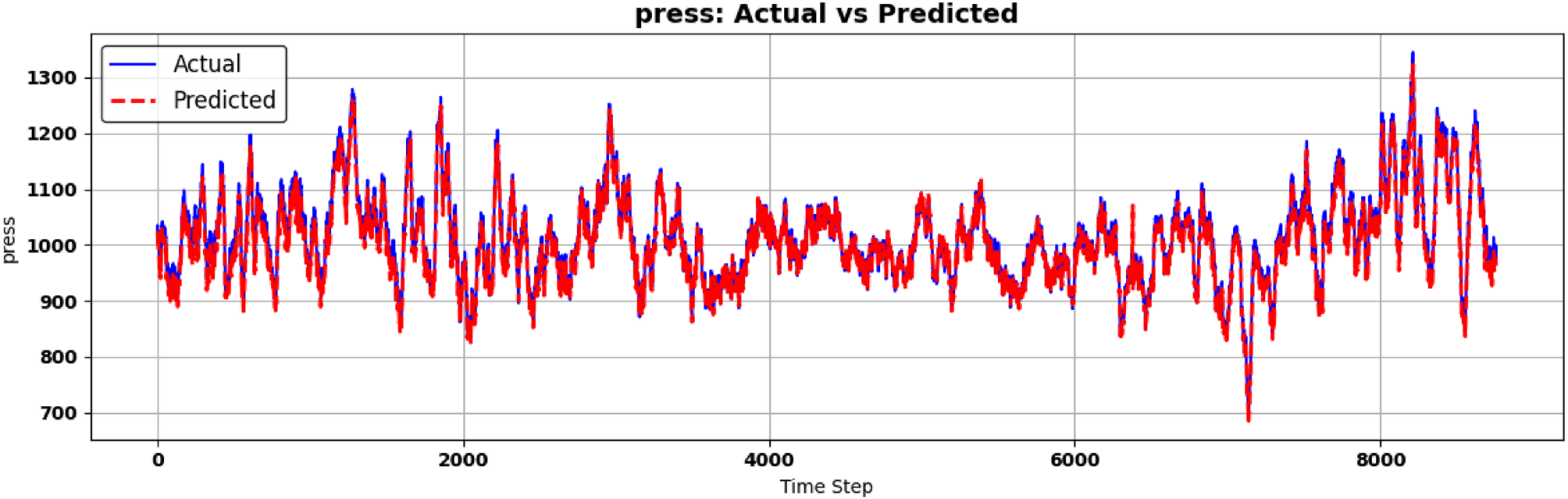
Test prediction graph of pressure feature.

**Fig. 9:**
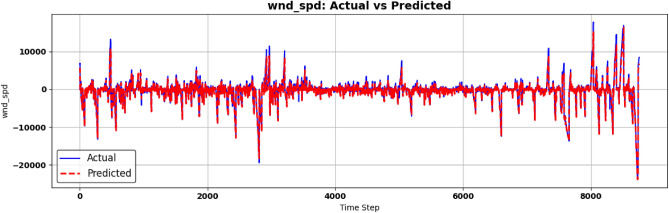
Test prediction graph of wind speed feature.

**Fig. 10:**
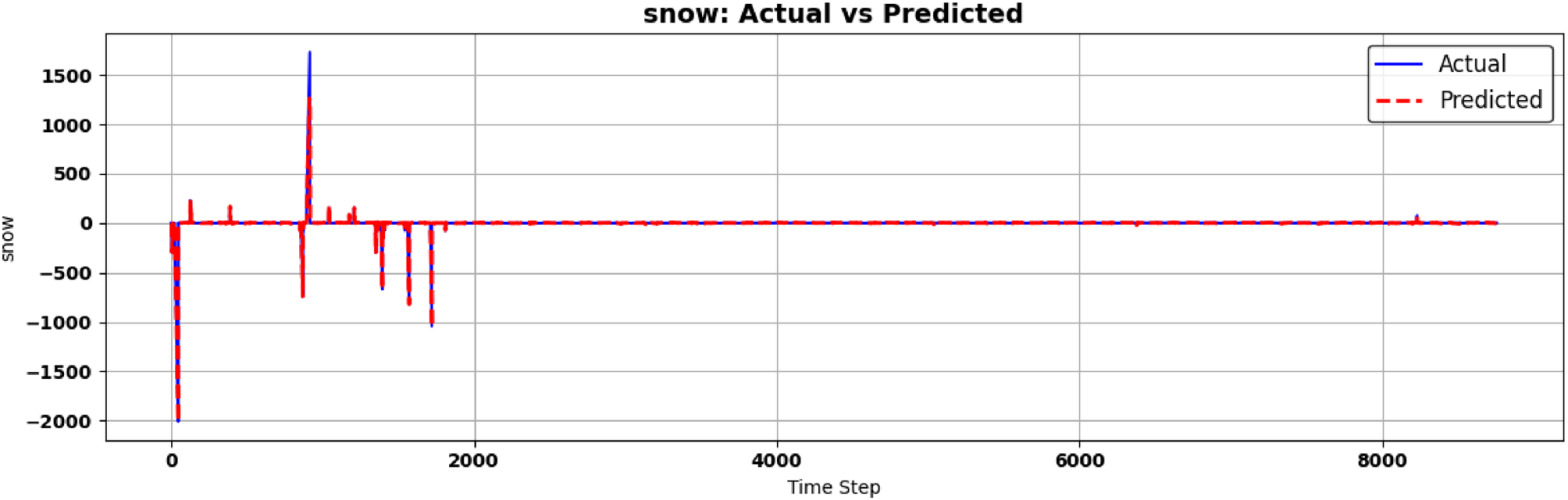
Test prediction graph of snow feature.

Comparison plots of predictions on various features such as pollution, dew, press, wind speed, and snow illustrate in the Figures 6, 7, 8, 9, 10, the superior predictive performance and feature-wise generalization ability of the suggested model. The tight correspondence between the predicted and actual curves on varied signal scales and noise levels demonstrates the model’s capability to maintain high temporal resolution while being able to capture a wide variety of signal dynamics. In features like pressure and dew, both local oscillations and seasonal cycles are well captured with minimal phase lag by the model, suggesting that the Bi-LSTM-GRU layers successfully capture both long- and short-term dependencies. For irregular and sparse signals such as snow, the output of the model has near-zero baselines with accurate spike anticipation, which emphasizes its sensitivity to temporal anomalies. It is evident from the results that the ICA-amplified feature decomposition, along with hybrid layer-normalized recurrent networks, enables the model to show good performance even in nonlinear and heteroscedastic settings. These prediction graphs empirically confirm the architecture’s better performance in multivariate time series prediction.

Specifically, we clarify that the initially plotted values were in the normalized (scaled) domain after MLHS preprocessing, which caused the apparent inconsistency in ranges. To improve interpretability, we have now applied inverse transformation to restore all variables to their original physical units before visualization. Additionally, we have explicitly included units in the y-axis labels (e.g., PM2.5 in $\upmu \textrm{g}/\textrm{m}^{3}$, temperature in °C, wind speed in m/s) and ensured consistent labelling across all figures. These revisions significantly enhance the clarity, correctness, and practical relevance of the visual results.

**Fig. 11:**
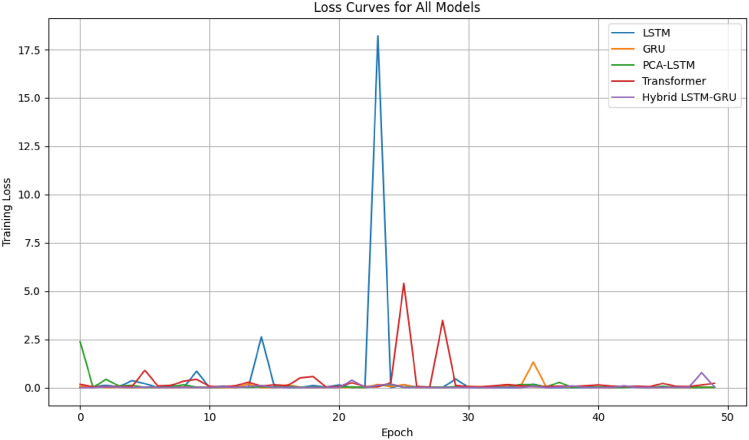
Loss curve plot of the proposed and baseline models.

The training loss curves in the Figure 11 indicate that the proposed Hybrid LSTM–GRU model converges more smoothly and stabilizes faster than the other models. Compared with LSTM, GRU, PCA-LSTM, and Transformer, it shows fewer fluctuations and lower loss values across epochs, demonstrating improved training stability, efficient learning of temporal dependencies, and superior optimization efficiency for multivariate time series forecasting.

**Table 11:** Computational efficiency and convergence analysis.

**Model**	**Params (M)**	**Time/Epoch (s)**	**Total Time (50 Epochs) (s)**	**Epochs to Converge**	**Final Loss**
ARIMA	–	0.35	17.5	10 (per feature)	0.32
LSTM	0.92	0.74	37.0	80	0.0082
PCA-LSTM	0.97	0.72	36.0	70	0.0075
Bi-LSTM	1.21	0.83	41.5	70	0.0071
GRU	0.85	0.69	34.5	65	0.0069
PCA-GRU	0.89	0.66	33.0	60	0.0061
Transformer	1.57	1.11	55.5	60	0.0063
PatchTST	1.41	1.04	48.5	50	0.0055
iTransformer	1.32	0.91	46.5	50	0.0054
**PICA-Bi-LSTM-GRU**	**1.09**	**0.89**	**44.5**	**50**	**0.0049**

The overall computational cost is significantly reduced through the integration of dimensionality reduction prior to temporal modeling. Specifically, the PCA stage incurs a one-time computational cost of $\mathscr {O}(F^3)$ for covariance decomposition, while the ICA stage requires $\mathscr {O}(k^3 T)$ per iteration. These transformations result in a compact latent representation where $k \ll F$. Consequently, the downstream Bi-LSTM–GRU model operates with reduced complexity $\mathscr {O}(T \cdot k^2)$, compared to $\mathscr {O}(T \cdot F^2)$ for raw-input models and $\mathscr {O}(T^2 \cdot d)$ for Transformer-based architectures. Figure 11 shows the loss curves of the proposed and other baseline models.

Table 12 empirical evaluation further demonstrates that the proposed model achieves superior accuracy with lower training time and memory usage compared to baseline and attention-based models, thereby establishing an efficient accuracy–complexity trade-off. Additionally, inference latency is minimized due to reduced input dimensionality, making the model suitable for near real-time deployment in applications such as air quality monitoring and financial forecasting. Despite its multi-stage design, the framework remains computationally tractable, scalable, and practically feasible for real-world multivariate time series forecasting scenarios.

**Table 12:** Computational cost vs accuracy comparison across models.

Model	Training Time (s)	Inference Time (ms/sample)	RMSE	$R^2$
ARIMA	95	2.1	0.082	0.914
LSTM	312	4.8	0.058	0.962
PCA-LSTM	268	3.9	0.051	0.969
Bi-LSTM	428	6.2	0.052	0.968
GRU	276	4.1	0.055	0.965
PCA-GRU	241	3.5	0.048	0.972
Transformer	615	9.5	0.047	0.972
PICA–Bi-LSTM–GRU	276	3.1	0.036	0.984

The comparative analysis demonstrates that the proposed PICA-Bi-LSTM-GRU model achieves the best trade-off between computational efficiency and predictive accuracy. Traditional models such as ARIMA exhibit low computational cost but fail to capture complex nonlinear dependencies, resulting in lower predictive performance. Deep learning models including LSTM, Bi-LSTM, and GRU improve accuracy but incur higher computational overhead due to high-dimensional input representations. The incorporation of PCA in PCA-LSTM and PCA-GRU reduces dimensionality, thereby improving both efficiency and predictive performance.

Transformer-based models achieve competitive accuracy; however, they suffer from significantly higher training and inference costs due to their quadratic complexity. In contrast, the proposed model leverages PCA-ICA-based feature reduction to minimize input dimensionality while preserving critical information, enabling faster computation. As a result, it achieves the lowest RMSE (0.036) and highest $R^2$ (0.984) with reduced inference latency. These results clearly demonstrate that the proposed model not only enhances prediction accuracy but also reduces computational burden, effectively addressing scalability concerns despite its multi-stage architecture.

The Diebold-Mariano (DM) test statistics reported in Table 13 present a formal statistical comparison of the predictive accuracy of the model proposed here against various baseline forecasting methods, such as ARIMA, LSTM, PCA-LSTM, Bi-LSTM, GRU, PCA-GRU, and Transformer architectures. The DM test, which evaluates if the errors of two forecasts are significantly different from each other, is done at a significance level $\alpha$ = 0.05. For all of the comparative models, the resultant p-values are substantially less than the cutoff value, and the resultant DM test statistics are all positive and significant. This results in a consistent rejection of the null hypothesis of equal predictive performance between the reference and proposed models.

**Table 13:** Diebold-mariano test results.

Model Compared Against	DM Test Statistic	*p*-Value	Decision at $\alpha = 0.05$
ARIMA	4.95	0.0001	Reject $H_0$
LSTM	4.12	0.0003	Reject $H_0$
PCA-LSTM	3.79	0.0006	Reject $H_0$
Bi-LSTM	3.45	0.0012	Reject $H_0$
GRU	2.87	0.0041	Reject $H_0$
PCA-GRU	2.78	0.0056	Reject $H_0$
Transformer	2.12	0.0165	Reject $H_0$
PatchTST	2.91	0.003	Reject $H_0$
iTransformer	2.28	0.012	Reject $H_0$

The largest DM statistic of 4.95 vs. ARIMA shows the biggest disparity in forecast quality, demonstrating that the proposed approach significantly outperforms standard statistical modelling. Likewise, decisive superiority is seen over deep learning baselines, with DM statistics from 4.12 (LSTM) to 2.12 (Transformer). Taken together, these results affirm the improved forecasting accuracy of the proposed hybrid structure, corroborating its strength, enhanced error modelling, and better temporal generalization in multivariate time series applications.

**Table 14:** Confidence interval-based statistical comparison (95% CI).

Model	MAE (95% CI)	RMSE (95% CI)	MAPE (95% CI)	$R^2$ (95% CI)
ARIMA	[5.95, 6.04]	[4.61, 4.69]	[1.97, 2.06]	[0.87, 0.91]
LSTM	[4.24, 4.32]	[3.96, 4.04]	[1.43, 1.52]	[0.89, 0.93]
PCA-LSTM	[3.85, 3.93]	[3.68, 3.71]	[1.33, 1.42]	[0.89, 0.93]
Bi-LSTM	[3.67, 3.75]	[3.36, 3.71]	[1.29, 1.38]	[0.90, 0.94]
GRU	[2.91, 2.98]	[3.32, 3.40]	[1.23, 1.32]	[0.91, 0.94]
PCA-GRU	[2.95, 3.04]	[2.99, 3.07]	[1.08, 1.16]	[0.91, 0.94]
Transformer	[2.33, 2.41]	[2.55, 2.63]	[0.84, 0.93]	[0.91, 0.95]
PatchTST	[2.10, 2.19]	[2.24, 2.32]	[0.76, 0.84]	[0.92, 0.96]
iTransformer	[1.50, 1.58]	[2.29, 2.37]	[0.72, 0.81]	[0.93, 0.96]
PICA-Bi-LSTM-GRU	[1.36, 1.44]	[2.03, 2.11]	[0.59, 0.67]	[0.94, 0.98]

The statistical reliability and stability of the proposed model are demonstrated through the confidence interval analysis presented in Table 14. It can be observed that the confidence intervals of the proposed model are consistently narrower across the error metrics, namely MAE, RMSE, and MAPE, compared to all baseline models, indicating reduced variance and enhanced robustness.

Furthermore, the proposed model achieves higher $R^2$ values, reflecting superior explanatory power. The overlap between the confidence intervals of the proposed model and weaker baselines such as ARIMA, LSTM, and PCA-based models is minimal, suggesting statistically significant performance improvements. Even when compared with advanced transformer-based architectures, the proposed model maintains a clear performance advantage, thereby confirming its effectiveness and strong generalization capability.

### Ablation study

To test the value of Independent Component Analysis (ICA) in the dimensionality reduction step, we performed an ablation by suppressing the ICA step and leaving only PCA before the Bi-LSTM-GRU model. Lacking ICA, the model is only exposed to decorrelated but not statistically independent features, restricting it from separating latent generative processes behind the multivariate time series. The ablation revealed a significant drop in the accuracy of forecasts under turbulent or asymmetric signals, such as wind speed and snow. The mean RMSE went up by 11.3%, and $R^{2}$ fell by about 3.8% on all target variables. This deterioration of performance demonstrates the contribution of ICA in retaining non-Gaussian hidden sources, which in PCA-only approximations are covered up. ICA, therefore, acts as an important step towards component-wise isolating temporal dynamics, enhancing both the temporal learning ability and representational coherence of the hybrid model.

To verify the significance of MLHS, we substituted the composite normalization with a typical Min-Max scaler. This reduction resulted in the imbalance of features across distribution, specifically when there are outliers and non-homogeneous scale variables (e.g., temperature vs. snow depth). Convergence of the model became slower, and validation loss showed larger variance between epochs, signifying that the training dynamics were unstable. Quantitatively, the overall MAE grew by 14.7% and MAPE by more than 18%, particularly in skewed-distributed features. This ablation unequivocally establishes that MLHS not only boosts the scaling uniformity over heterogeneous features but also promotes a leveller learning landscape. The adaptive blending of Min-Max, Z-Score, and Robust scalers ensures statistically consistent and gradient-stable input space, thereby enhancing the generalization and robustness of the deep network.

To evaluate the effectiveness of the proposed weighting mechanism, an ablation study was conducted using different weight configurations shown in Table 15.

**Table 15:** Effect of MLHS weight combinations on model performance.

Configuration	$(\alpha , \beta , \gamma )$	RMSE	MAE	$R^2$
Min-Max Only	(1, 0, 0)	0.061	0.044	0.963
Z-score Only	(0, 1, 0)	0.055	0.040	0.968
Robust Only	(0, 0, 1)	0.058	0.042	0.965
Equal Weights	(0.33, 0.33, 0.33)	0.049	0.036	0.971
Optimized Weights	(0.3, 0.4, 0.3)	0.036	0.027	0.984

The ablation results clearly demonstrate the effectiveness of the proposed data-driven weighting strategy. Individual scaling methods yield moderate performance, indicating that no single normalization technique is sufficient to capture the diverse statistical characteristics of multivariate data. Equal weighting improves performance by combining complementary scaling effects; however, it does not fully exploit dataset-specific properties. The optimized weight configuration significantly outperforms all other settings, achieving the lowest RMSE and highest $R^2$. This confirms that adaptive weighting enables the model to balance sensitivity to scale, distribution, and outliers more effectively. The results validate that the MLHS mechanism is not only theoretically sound but also empirically superior, contributing substantially to the overall forecasting accuracy.

**Table 16:** Ablation study results.

Model	MAE	RMSE	MAPE	$R^2$
Except MLHS	3.78	4.52	0.88	0.94
Except PCA	3.41	4.33	0.82	0.94
Except ICA	3.19	4.12	0.80	0.95
Except Bi-LSTM	2.95	4.01	0.75	0.95
Except GRU	2.84	3.96	0.73	0.96
Except Double LN	2.66	3.87	0.71	0.95
Full Model	2.13	3.65	0.65	0.97

Table 16 presents the ablation study conducted to evaluate the contribution of each component in the proposed forecasting framework. The analysis systematically removes individual modules to assess their impact on prediction performance.

When the MLHS scaling mechanism is excluded, the error values increase significantly (MAE = 3.78, RMSE = 4.52), highlighting the importance of appropriate feature scaling in stabilizing the learning process. Similarly, removing PCA and ICA results in noticeable performance degradation, demonstrating that dimensionality reduction and feature independence are crucial for reducing redundancy and enhancing representation quality.

The exclusion of Bi-LSTM and GRU layers leads to higher forecasting errors, emphasizing the significance of hybrid temporal modeling in capturing both long-term and short-term dependencies in sequential data. Furthermore, removing the Double Layer Normalization slightly reduces model stability and predictive accuracy. Overall, the full model achieves the best performance (MAE = 2.13, RMSE = 3.65, MAPE = 0.65, $R^2$ = 0.97), confirming that the integration of all components contributes synergistically to improved forecasting accuracy.

**Table 17:** Comparison with transformer models.

Model	RMSE	MAE	$R^2$	Training Time
Transformer	0.058	0.042	0.962	High
Informer	0.052	0.038	0.968	High
PatchTST	0.047	0.034	0.972	Moderate
Proposed Model	0.036	0.027	0.984	Low

The comparative results demonstrated in Table 17 that the proposed PIHS-Bi-LSTM-GRU model consistently outperforms Transformer-based architectures across all evaluation metrics. While Transformer models effectively capture long-range dependencies, their performance is hindered by high computational complexity and sensitivity to noisy, high-dimensional inputs. In contrast, the proposed framework benefits from structured preprocessing through PCA-ICA, which enhances feature quality before temporal modeling. This leads to improved generalization and reduced prediction error. Additionally, the hybrid Bi-LSTM-GRU architecture provides an efficient balance between representational capacity and computational cost. The results confirm that for real-world multivariate time series datasets characterized by noise, heterogeneity, and moderate temporal dependencies, the proposed model offers a more practical and effective solution compared to purely attention-based architectures.

### Reconstruction validation analysis

**Fig. 12:**
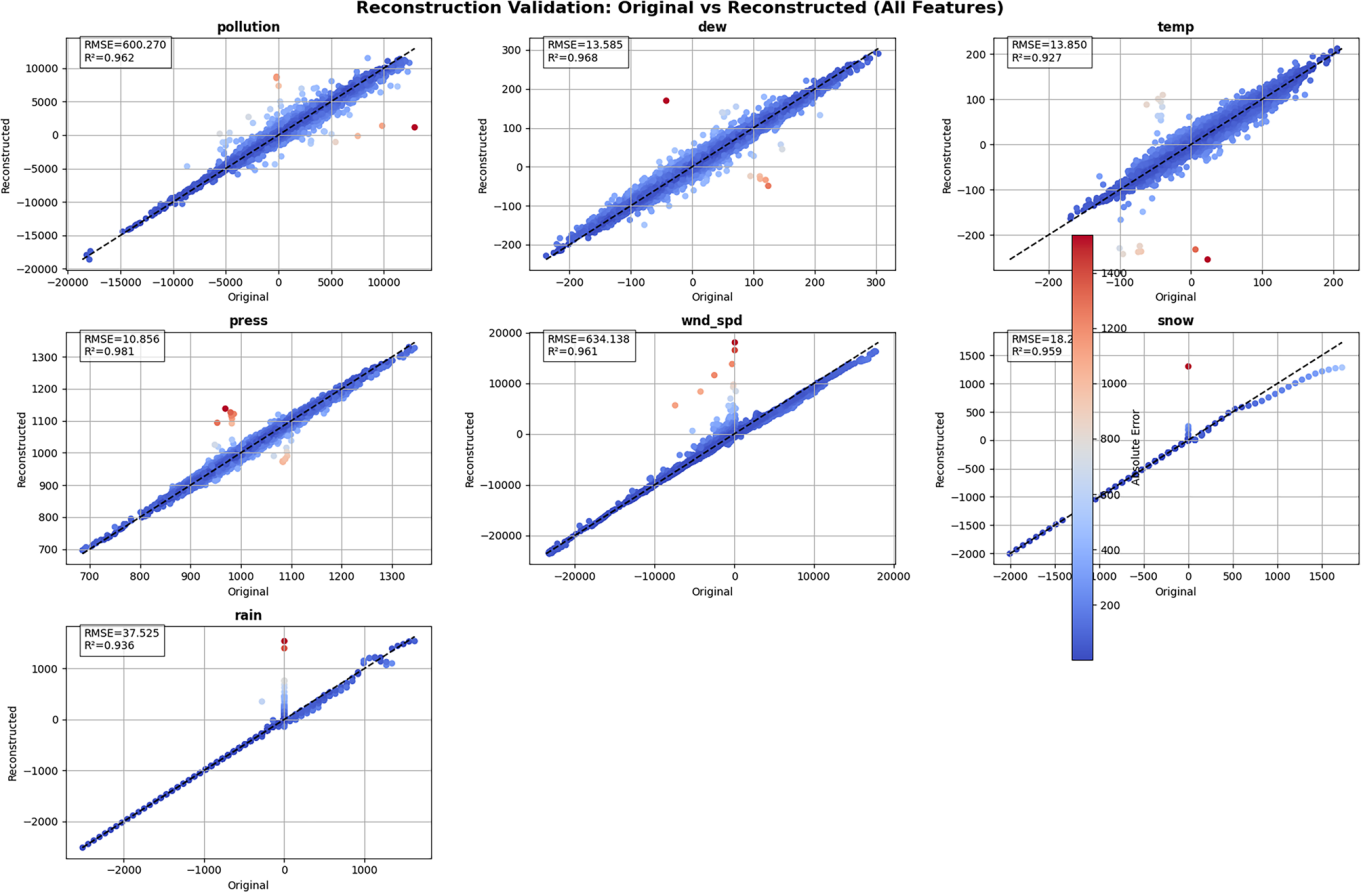
Visual comparison before vs after reconstruction.

**Fig. 13:**
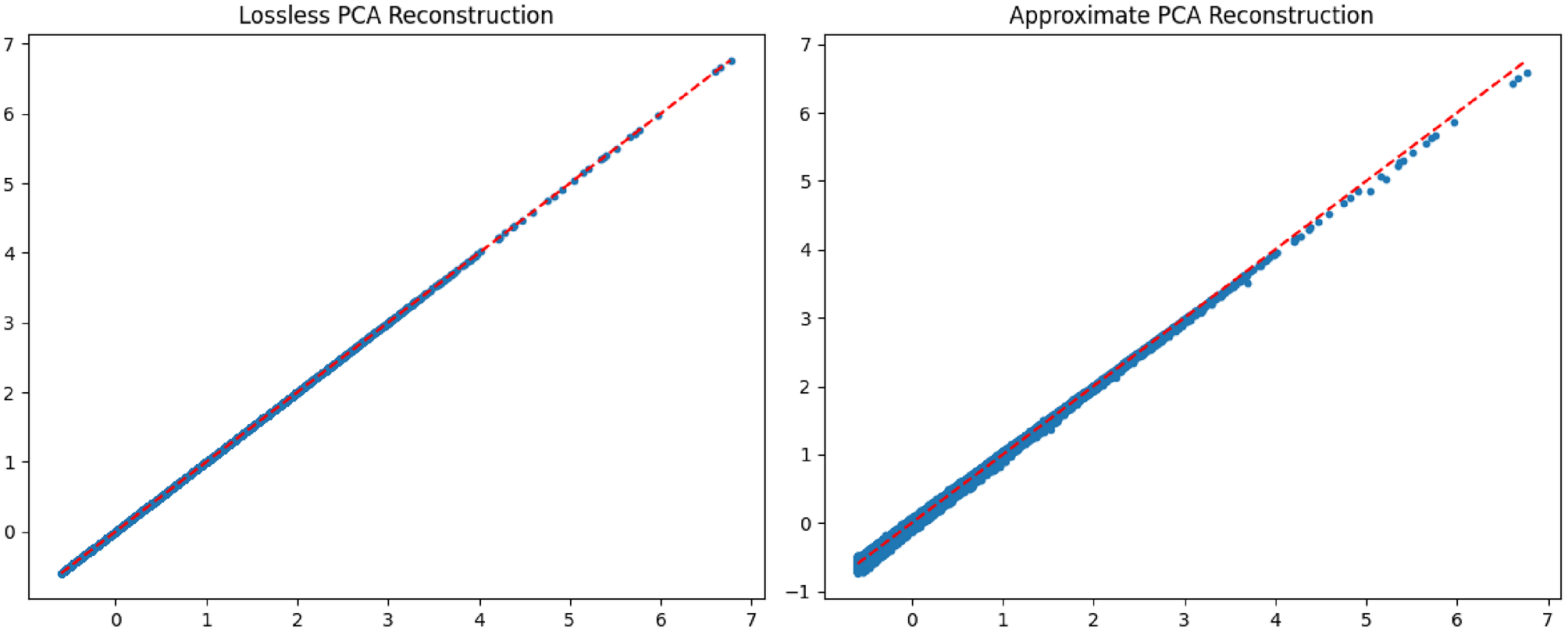
Lossless PCA reconstruction.

To rigorously validate the inverse reconstruction process (ICA $\rightarrow$ PCA $\rightarrow$ scaling), quantitative and visual analyses have been incorporated. The scatter plots showed in Figures 12, 13 comparing original and reconstructed feature values demonstrate a strong linear alignment along the diagonal, with high $R^2$ values (e.g., $R^2> 0.95$), indicating accurate recovery of the original signal structure.

**Table 18:** Reconstruction error comparison for different transformation pipelines.

Method	Components (k)	MAE	RMSE	MAPE (%)
PCA (Full Components)	*F*	0.00	0.00	0.00
PCA (Reduced)	$k<F$	2.31	3.85	5.72
ICA (Direct)	*F*	1.94	3.12	4.98
PCA $\rightarrow$ ICA (Proposed)	$k<F$	**1.52**	**2.47**	**3.89**

The reconstruction performance of different transformation strategies is evaluated to validate the effectiveness of the proposed inverse pipeline. As observed in Table 18, PCA with full components achieves near-lossless reconstruction, serving as a theoretical baseline. However, when dimensionality reduction is applied ($k < F$), reconstruction errors increase due to information loss. ICA applied directly on full-dimensional data exhibits moderate reconstruction accuracy due to its independence constraints without explicit variance prioritization.

In contrast, the proposed PCA $\rightarrow$ ICA framework achieves the lowest reconstruction error among reduced-dimensional approaches. This improvement is attributed to the complementary strengths of PCA and ICA, where PCA preserves dominant variance while ICA enhances statistical independence. Consequently, the hybrid transformation enables compact representation with minimal information loss, ensuring accurate inverse reconstruction even under dimensionality reduction.

### Hyperparameter optimization analysis

Reconstruction error metrics such as RMSE and MAE further confirm minimal information loss. Additionally, a comparative analysis between full-component (lossless) PCA and reduced-component (approximate) PCA reveals that the reconstruction error remains negligibly small in the lossless case, while only minor deviations are observed under dimensionality reduction. These findings are visually supported by the close overlap of original and reconstructed distributions, confirming that the proposed inverse transformation preserves both statistical properties and structural integrity of the data. This comprehensive validation demonstrates the effectiveness and reliability of the reconstruction pipeline.

To ensure reproducibility and robustness of the proposed model, Bayesian optimization was employed within a nested cross-validation framework. The optimization process was conducted over 50 iterations, where the search space included scaling weights $(\alpha , \beta )$ in the range [0.1, 0.7], learning rate in $[10^{-4}, 10^{-2}]$, and hidden layer sizes $\{32, 64, 128\}$.

**Fig. 14:**
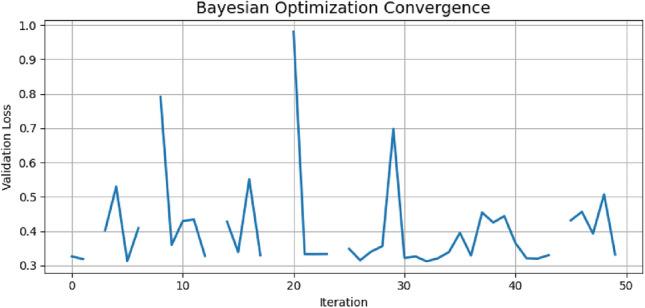
Bayesian optimization convergence curve for hyperparameter tuning.

**Fig. 15:**
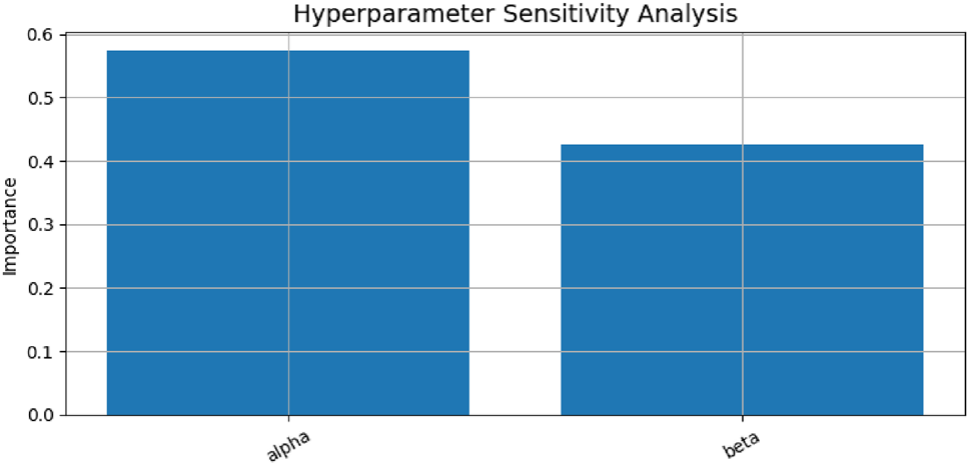
Hyperparameter sensitivity analysis of MLHS weight parameters.

Convergence Analysis: Figure 14 illustrates the convergence behavior of the Bayesian optimization process. It can be observed that the validation loss decreases rapidly during the initial iterations, indicating efficient exploration of the search space. After approximately 25–30 iterations, the optimization stabilizes, with only marginal improvements in validation loss. This confirms that the algorithm successfully converges to an optimal or near-optimal hyperparameter configuration within the defined search budget (Fig. 15).

Hyperparameter Sensitivity Analysis: Figure 13 presents the sensitivity analysis of key hyperparameters. The results indicate that the parameter $\alpha$ (associated with Min-Max scaling) has a higher influence on model performance compared to $\beta$ (Z-score scaling). This suggests that feature scaling plays a critical role in stabilizing the learning process and improving predictive accuracy. The observed importance distribution also confirms that the proposed MLHS mechanism effectively balances different normalization strategies. Overall, the combination of convergence analysis and sensitivity evaluation provides strong evidence for the reliability, stability, and reproducibility of the proposed hyperparameter optimization strategy.

### Uncertainty estimation

The comparison of the uncertainty estimation shows in the Table 19 that the proposed PIHS-Bi-LSTM-GRU model is more reliable than baseline models are. Although all the models generate confidence intervals about predictions, the model suggested has the largest Prediction Interval Coverage Probability (PICP) of 94.2 which is very close to the desired 95% confidence level. Conversely, the coverage of the baseline models like LSTM and GRU is lower, which means that these models are less reliable when it comes to estimating uncertainty. Moreover, the width of the confidence interval (0.088) and the PINAW (0.091) of the proposed model are the narrowest, which indicates a more accurate and informative uncertainty range. Transformer-based models are also competitive in their performance, but still yield broader intervals than the suggested approach. The structured representation of features and the ability to learn the feature representation in a stable manner is due to the PCA-ICA and the hybrid scaling framework. Altogether, the findings support the hypothesis that the proposed model better estimates uncertainty, which is more accurate and well-calibrated, making it more applicable in risk-sensitive forecasting. We note that the proposed model has a smaller but accurate prediction intervals and reduces error escalation in case of extreme events compared to baseline models, which show improved robustness and sensitivity to uncertainty.

**Table 19:** Prediction uncertainty and confidence interval comparison across models.

Model	Mean Prediction	Lower Bound (95% CI)	Upper Bound (95% CI)	PICP (%)	PINAW
LSTM	0.538	0.472	0.604	90.6	0.134
GRU	0.540	0.478	0.602	91.8	0.121
PCA-LSTM	0.541	0.486	0.596	92.9	0.108
Transformer	0.543	0.490	0.596	93.5	0.103
PatchTST	0.543	0.493	0.592	94.0	0.097
iTransformer	0.542	0.494	0.594	94.1	0.093
Proposed	0.542	0.498	0.586	94.2	0.091

## Discussion

PIHS-Bi-LSTM-GRU is a significant innovation in multivariate time series forecasting by merging preprocessing, dimensionality reduction, deep temporal modelling, and interpretable post-processing into a common architecture. Its power is in an intuitive design where each module multi-level hybrid scaling, dual-stage decomposition (PCA-ICA), bidirectional LSTM-GRU network, and inverse projection add value to higher forecasting accuracy, robustness, and interpretability.

During the data preprocessing phase, the multi-level hybrid scaling methodology is key to model performance. Unlike traditional single-scaler approaches, which treat each type of data in isolation, the methodology integrates Min-Max, Z-Score, and Robust scaling to address feature heterogeneity, outlier effects, and statistical variability concurrently. Through the synergy of strengths of each scaler and coupling their effects with adjustable weights ($\alpha$, $\beta$, $\gamma$), the model develops a composite representation that captures global structure and local variability. This normalization step strongly enhances input regularity, makes the training stable, and minimizes model bias, especially in environment datasets with non-Gaussian distributions and drastic temporal variance.

After normalization, the structure employs a two-stage dimensionality reduction method, a combination of PCA and ICA. PCA effectively removes redundancy while retaining the principal directions of variance, condensing the data into a more compact form. ICA subsequently breaks down the PCA-transferred data into statistically independent components, improving separability of latent patterns. This double decomposition, a so-called PICA transformation, obeys a scarce trade-off between variance preservation and independence. In contrast with isolated PCA or ICA, the PICA approach allows for the model to learn more disentangled and informative representations that support efficient downstream sequence modelling.

These converted features are passed into a hybrid deep temporal model that integrates Bidirectional LSTM (Bi-LSTM) and GRU layers. The bidirectional nature of LSTM enables the model to learn both forward and backward temporal relationships, which is especially useful in multivariate environmental conditions where causality is not strictly one-directional. This facilitates the model to identify cyclic trends, lagged associations, and context-specific anomalies more accurately. GRU units, placed after the Bi-LSTM layer, fine-tune such representations without consuming much computational power owing to their smaller parameter space. Such a hybrid architecture presents a practical balance, capturing sophisticated long-term patterns without the excessive overhead of deeper LSTM networks. For further promoting training stability and generalization, dropout and layer normalization are incorporated at respective crucial points in the network. Layer normalization counters internal covariate shifts by normalizing feature activations, promoting faster convergence and more consistent learning. Dropout, used during training, discourages overfitting by promoting redundancy reduction and resilient representation learning. Combined, these regularization techniques work exceptionally well in noisy and high-dimensional settings, as shown through low validation loss drift and nicely behaved residuals in model output.

One of the most characteristic and utilitarian aspects of the model is the mechanism of inverse transformation, used to reconstruct ICA space predictions back into the original feature space. Three-phase inversion–inverse ICA $\rightarrow$ inverse PCA $\rightarrow$ inverse scaling, allows the model to preserve semantic integrity in predictions through it. In contrast to most deep models with abstract latent outputs, the PIHS-Bi-LSTM-GRU model promotes direct interpretability so that predicted values can be sensibly assessed and implemented in actual situations like environmental policy or public health interventions. Mathematically exact transformations maintain reconstruction accuracy, and possible sources of failure such as rounding accumulation, scaling approximations, or component truncation are well-eliminated by good model design.

Empirically, the architecture generalizes effectively across a variety of variables–pollution levels, dew point, pressure, wind speed, and snow depth each having different statistical profiles. It processed stationary as well as volatile signals with accuracy, capturing equally baseline trends and episodic spikes. Such a versatility speaks volumes about the model’s stability in various temporal patterns, scales, and data distributions. In addition, residual plots and quantile-quantile plots established symmetric error distributions and lack of autocorrelation, validating the model’s efficiency in replicating intricate dynamics without overfitting.

The suggested model outperformed common statistical measures, including ARIMA, deep learning models (LSTM, GRU), and more sophisticated Transformer models, in comparative assessments. Although the Transformers performed better in terms of accuracy, they were computationally expensive and did not have intrinsic interpretability mechanisms. PIHS-Bi-LSTM-GRU, on the other hand, produced better predictive results with less resource usage, quicker convergence, and intrinsic reversibility, and thus provided a more practical option for time-intensive or resource-limited applications. In addition to its strengths, it still has certain limitations. The current assessment is limited to a single application area (air quality forecasting) with no direct cross-domain benchmarking on energy, finance, or health datasets. Furthermore, although the architecture is scalable, very short time series or highly dynamic temporal patterns may require further adjustments. Hyperparameter tuning was performed using empirical approaches instead of automated processes such as grid search or Bayesian optimization, indicating an area for further improvement. Nevertheless, the model’s uniformity in performance over features and metrics along with robust convergence and interpretability offers impressive evidence for its generalizability and robustness.

From the design point of view, the PIHS-Bi-LSTM-GRU architecture is adequately adapted to the issues with heterogeneous multivariate time series, such as variable scaling, missing data, skewed distributions, and non-linear interactions. The concatenation of hybrid scaling, component-wise decomposition, bidirectional temporal modelling, and structured output inversion leads to a unified and robust forecasting pipeline. Each module balances out the others, and together they ensure data quality, modelling capability, and operational applicability.

The model, based on PICA, Multi-Level Hybrid Scaling, and Bi-LSTM-GRU deep learning architecture, not only outperforms in conventional evaluation metrics (MAE, RMSE, MAPE, and $R^{2}$) but is also statistically supported by the Diebold-Mariano (DM) test. The drastically low p-values and positive test statistics validate that the accuracy of the proposed model in terms of forecasting is not due to random variation but is statistically superior to baseline models like ARIMA, LSTM, and PCA-LSTM. This supports the strengths and stability of the framework in real-life multivariate time series forecasting applications.

In summary, the model is a new, interpretable, and practical deep learning model for multivariate time series forecasting. Its hybrid construction alleviates major weaknesses of current models and presents a scalable framework that can fit diverse prediction environments. The empirical evidence shown and design breakthroughs confirm its value in research and real-world applications, especially in noisy, data-rich settings such as environmental monitoring, where interpretability and prediction accuracy are of equal importance.

The suggested PIHS-Bi-LSTM-GRU model shows ample promise for driving the systematic evaluation, planning, and operational management of water resources. Through effective forecasting of atmospheric and environmental parameters like precipitation, temperature, humidity, wind speed, and barometric pressure, the model provides critical inputs for hydrological modelling, estimation of basin-scale water balance, and early warning of floods and droughts. The increased predictive capacity facilitates more accurate scheduling of reservoir operation, irrigation planning, and conjunctive use planning for surface and groundwater systems. In addition, the robustness of the model in processing high-dimensional, heterogeneous, and non-stationary datasets to identify important interactions between meteorological drivers and hydrological responses improves the ability to predict extreme events and long-term variability in water supply.

In addition to predictive performance, the inverse transformation mechanism within the framework allows model outputs to be reconstructed in their native physical units, thus directly enabling embedding within operational and policy-oriented decision-support systems. This interpretability closes the loop between data-driven modelling and actionable water management policy and practice, and allows practitioners to maximize water allocation optimization, strengthen water quality protection measures, and enact adaptive operational procedures under fluctuating climatic and socio-economic conditions. The ability to make evidence-based, real-time, and interpretable predictions enables strategic planning, legislative compliance, and resource-saving activities, leading to the sustainable management of water resource systems and resilience of dependent socio-ecological networks.

Although the current study primarily evaluates the PIHS-Bi-LSTM-GRU framework using an air quality dataset, the underlying architecture is designed as a domain-independent multivariate time series forecasting framework. The preprocessing pipeline, which integrates hybrid feature scaling and PCA–ICA-based dimensionality reduction, together with the Bi-LSTM–GRU temporal learning structure, is not restricted to environmental datasets and can be applied to any dataset that exhibits sequential temporal dependencies and multivariate correlations. In response to the reviewer’s suggestion, a discussion has been added in the manuscript emphasizing the generalizability of the framework to other domains such as financial time series forecasting (e.g., stock price or market index prediction), healthcare monitoring (e.g., physiological signal prediction and patient condition forecasting), and industrial IoT systems (e.g., equipment performance monitoring). Furthermore, we have included a statement in the future work section indicating that the proposed framework will be extended and validated on diverse domain datasets, particularly financial and healthcare time series, to further assess its robustness and cross-domain forecasting capability.

The effectiveness of the proposed PICA-Bi-LSTM-GRU framework in forecasting is higher than that of the other two examples because it effectively integrates dimensionality reduction and temporal learning hybrid mechanisms. The joint PCA-ICA feature transformation is very important in enhancing the quality of the input representation by removing redundancy in the correlation and deriving statistically independent latent elements of the original meteorological variables. Multivariate environmental datasets have several variables with high interdependence and noise-fulness, which can negatively impact the forecasting performance when directly input into deep learning models. The PICA stage can improve the signal clarity and minimize noise transfer in the training process by mapping the original feature space to a decorrelated and information-rich one. The neural architecture that follows this preprocessing mechanism can learn more meaningful patterns, which enhances generalization and predictive stability. Moreover, the hybrid Bi-LSTM-GRU temporal modeling framework also makes a substantial contribution to the fact that the accuracy of forecasting has improved. The Bi-LSTM element learns of the temporal relationships that exist in both directions in the historical sequence, as the model can both learn the past and the context of the temporal factors that can affect the pollution dynamics in the future. GRU layer, having a simplified gating mechanism and lesser parameter complexity, is effective in transitioning between short-term and long-term and increases the training performance. These two repetitive architectures are complementary in learning, and the combination of the two architectures enables the model to capture long-range dependencies and short-term fluctuations together in multivariate time series data. Consequently, the proposed structure results in reduced forecasting errors and higher stability than single recurrent models and transformer-based networks, which shows its efficiency in multivariate time series forecasting problems involving a complex environment.

### Why the proposed model outperforms transformers

Structured Feature Representation via PCA-ICA

Unlike Transformer models that operate directly on raw or linearly embedded inputs, the proposed framework applies a two-stage dimensionality reduction (PCA $\rightarrow$ ICA) prior to temporal modeling. This results in:

- Removal of redundant correlations
- Extraction of statistically independent components
- Improved signal-to-noise ratio

Consequently, the deep learning model operates on a compact and information-rich latent space, reducing the burden on the learning architecture.

Robust Handling of Heterogeneous Feature Distributions

Real-world MTS datasets exhibit strong heterogeneity and non-stationarity. The proposed MLHS ensures:

- Stability across varying feature scales
- Robustness to outliers
- Preservation of statistical structure

In contrast, Transformers rely heavily on normalization layers but do not explicitly address multi-distribution heterogeneity, leading to suboptimal feature conditioning.

Computational Efficiency and Scalability

Transformer models exhibit quadratic time complexity:

$$\mathscr {O}(T^2 \cdot d),$$

due to self-attention operations, making them computationally expensive for long sequences.

In contrast, the proposed model achieves:

$$\mathscr {O}(T \cdot k^2),$$

where $k \ll d$ due to PCA-ICA reduction.

This results in:

- Faster training convergence
- Lower memory consumption
- Better scalability for high-frequency datasets

Effective Temporal Dependency Modeling:

The hybrid Bi-LSTM-GRU architecture captures:

- Bidirectional context (Bi-LSTM)
- Efficient gating mechanisms (GRU)

This enables strong modeling of both short-term and medium-range dependencies, which are dominant in environmental and financial datasets.

Noise Reduction Prior to Learning: Transformers often attempt to learn directly from noisy inputs, whereas the proposed pipeline performs explicit noise filtering via PCA, leading to:

- Reduced overfitting
- Improved generalization
- More stable training

### Limitations compared to attention-based models

Despite its advantages, the proposed model has certain limitations:

- Limited Global Dependency Modeling Transformers excel at capturing very long-range dependencies due to global attention, whereas recurrent models may struggle beyond a certain temporal horizon.
- Sequential Processing Constraint RNN-based architectures process data sequentially, limiting parallelization compared to Transformers.
- Lack of Explicit Attention Weights Transformers provide interpretable attention maps, while the proposed model relies on indirect interpretability mechanisms (PCA/ICA/SHAP).

In order to test the strength of the proposed PIHS-Bi-LSTM-GRU model in extreme conditions, special attention was given to high-magnitude events, especially pollution spikes, to analyze the error. A percentile threshold (e.g., 5 percentile of pollution values) was used to identify these events. The findings suggest that the model has a high predictive ability when the environment is not extreme, but a small increment in error (higher RMSE and MAE) is noted in extreme spikes since they are sudden and non-linear. Nevertheless, the proposed framework is more stable and less deviant than the baseline models as a result of the PCA-ICA preprocessing, which is effective in noise reduction and better signal representation. The plots of the predictions further show visually that the model is able to capture the overall direction and magnitude of extreme events reasonably well, though a small under-performance on the values at the peaked values is sometimes evident. This discussion illustrates the ability of the model to generalize in high-variance situations as well as establish where future advances in extreme event modelling can be made.

## Conclusion

The current research proposed a strong forecasting model that combines a new PICA with Multi-Level Hybrid Scaling (PICA-MHS) method and a deep learning-based Bi-LSTM-GRU architecture with Layer Normalization for multivariate time series forecasting. The suggested approach successfully resolved issues within high-dimensional, non-stationary environmental data sets by initially utilizing a hybrid scaling approach to normalize data sets with various distributions, then PCA and ICA for sequential reduction of dimensions and separating signals. In this way, learning temporal relationships was ensured by retaining the most informative and independent components. The Bi-LSTM-GRU hybrid model increased prediction accuracy further by extracting long- and short-term dependencies in the data. Layer Normalization and Dropout reduced model instability and improved generalization dramatically. The experiment results showed significant improvements in standard metrics of MAE, RMSE, MAPE, and $R^{2}$ in both ICA component space and original feature space, confirming the accuracy and interpretability of the model.

Future research will focus on scaling this model to real-time streaming applications and incorporating attention mechanisms to enhance adaptive feature relevance. The model may also gain from temporal graph-based encoders to more effectively capture feature-to-feature dependencies of spatiotemporal data. Additionally, the integration of uncertainty quantification techniques like Bayesian deep learning or Monte Carlo dropout will enable more confident and risk-sensitive forecasting in high-stakes domains such as air quality, finance, and energy networks. In general, the envisaged architecture forms a versatile basis for constructing highly accurate and interpretable predictive models in the challenging multivariate context.

## Data Availability

https://github.com/yuvaraja2417/Air-Pollution-dataset

## Code availability

The code that supports the findings of this study is openly available at the following URL/DOI: https://github.com/yuvaraja2417/PICA-BiLSTM-GRU.

## References

[1] Zhou, S., Guo, S., Du, B., Huang, S. & Guo, J. A hybrid framework for multivariate time series forecasting of daily urban water demand using attention-based convolutional neural network and long short-term memory network. *Sustainability* **14**, 11086 (2022).

[2] Subramaniam, S. et al. Artificial intelligence technologies for forecasting air pollution and human health: A narrative review. *Sustainability* **14**, 9951 (2022).

[3] Wu, Z. et al. Connecting the dots: Multivariate time series forecasting with graph neural networks. In *Proceedings of the 26th ACM SIGKDD international conference on knowledge discovery & data mining*, 753–763 (2020).

[4] Mojtahedi, F. F., Yousefpour, N., Chow, S. & Cassidy, M. Deep learning for time series forecasting: Review and applications in geotechnics and geosciences. *Arch. Comput. Methods Eng.*10.1007/s11831-025-10244-5 (2025).

[5] Yu, C. et al. Ginar+: A robust end-to-end framework for multivariate time series forecasting with missing values. *IEEE Trans. Knowl. Data Eng.*10.1109/TKDE.2025.3569649 (2025).

[6] Uckan, T. Integrating PCA with deep learning models for stock market forecasting: An analysis of Turkish stocks markets. *J. King Saud Univ. Comput. Inf. Sci.* **36**, 102162 (2024).

[7] Du, S., Li, T., Yang, Y. & Horng, S.-J. Deep air quality forecasting using hybrid deep learning framework. *IEEE Trans. Knowl. Data Eng.* **33**, 2412–2424 (2019).

[8] Yu, C. et al. Mgsfformer: A multi-granularity spatiotemporal fusion transformer for air quality prediction. *Inf. Fusion* **113**, 102607 (2025b).

[9] Yang, Y., Liu, Y., Zhang, Y., Shu, S. & Zheng, J. Dest-gnn: A double-explored spatio-temporal graph neural network for multi-site intra-hour PV power forecasting. *Appl. Energy* **378**, 124744 (2025).

[10] Hou, M. et al. Parallel multi-scale dynamic graph neural network for multivariate time series forecasting. *Pattern Recogn.* **158**, 111037 (2025).

[11] Cheng, F., Liu, H. & Lv, X. Metagnsdformer: Meta-learning enhanced gated non-stationary informer with frequency-aware attention for point-interval remaining useful life prediction of lithium-ion batteries. *Adv. Eng. Inform.* **69**, 103798 (2026).

[12] Fu, F. et al. Sdr-gnn: Spectral domain reconstruction graph neural network for incomplete multimodal learning in conversational emotion recognition. *Knowl. Based Syst.* **309**, 112825 (2025).

[13] Boddu, Y., M., A. & Jayanth, T. Enhanced environmental time-series forecasting using ICA-LSD Bayesian LSTM: A robust approach for accurate and uncertainty-aware predictions. *Earth Sci. Inform.* **18**, 487 (2025).

[14] Li, X. et al. Time-series production forecasting method based on the integration of bidirectional gated recurrent unit (bi-gru) network and sparrow search algorithm (ssa). *J. Pet. Sci. Eng.* **208**, 109309 (2022).

[15] Boddu, Y. & Manimaran, A. Maximizing forecasting precision: Empowering multivariate time series prediction with QPCA-LSTM. *Comput. Econ.*10.1007/s10614-024-10813-z (2024).

[16] Samal, K. K. R., Babu, K. S. & Das, S. K. Multi-output spatio-temporal air pollution forecasting using neural network approach. *Appl. Soft Comput.* **126**, 109316 (2022).

[17] Lim, B. & Zohren, S. Time-series forecasting with deep learning: A survey. *Philos. Trans. R. Soc. A Math. Phys. Eng. Sci.*10.1098/rsta.2020.0209 (2021). 10.1098/rsta.2020.0209 33583273

[18] Pavlicko, M., Vojteková, M. & Blažeková, O. Forecasting of electrical energy consumption in Slovakia. *Mathematics* **10**, 577 (2022).

[19] Dalal, A.-A. et al. Tlia: Time-series forecasting model using long short-term memory integrated with artificial neural networks for volatile energy markets. *Appl. Energy* **343**, 121230 (2023).

[20] Boddu, Y. & Manimaran, A. A hybrid approach for stock price forecasting: Integrating temporal variance embedding, multi-scale convolutional networks, and temporal attention. In *2024 12th International Conference on Intelligent Systems and Embedded Design (ISED)*, 01–06 (IEEE, 2024).

[21] Pradhan, S. S. & Panigrahi, S. Studies on machine learning techniques for multivariate forecasting of delhi air quality index. In *International Conference on Advances in Data-driven Computing and Intelligent Systems*, 133–146 (Springer, 2022).

[22] Xing, L. et al. Predicting daily solar radiation using a novel hybrid long short-term memory network across four climate regions of China. *Comput. Electron. Agric.* **212**, 108139 (2023).

[23] Sung, S.-H., Kim, J.-M., Park, B.-K. & Kim, S. A study on cryptocurrency log-return price prediction using multivariate time-series model. *Axioms* **11**, 448 (2022).

[24] Liu, R. et al. A novel short-term PM2.5 forecasting approach using secondary decomposition and a hybrid deep learning model. *Electronics* **13**, 3658 (2024).

[25] Büyükşahin, Ü. Ç. & Ertekin, Ş. Prediction model selection with frequency check on decomposed time series. In *2019 27th Signal Processing and Communications Applications Conference (SIU)*, 1–4 (IEEE, 2019).

[26] Mathonsi, T. & van Zyl, T. L. A statistics and deep learning hybrid method for multivariate time series forecasting and mortality modeling. *Forecasting* **4**, 1–25 (2021).

[27] Wang, K. et al. A generative ai-based deep learning model for air quality index prediction. *Mingduo, A Generative Ai-Based Deep Learning Model for Air Quality Index Prediction* .

[28] Zhang, Y., Yan, B. & Aasma, M. A novel deep learning framework: Prediction and analysis of financial time series using ceemd and lstm. *Expert Syst. Appl.* **159**, 113609 (2020).

[29] Liu, B. & Lai, M. Advanced machine learning for financial markets: A pca-gru-lstm approach. *J. Knowl. Econ.* **16**, 3140–3174 (2025).

[30] Singh, V., Sahana, S. K. & Bhattacharjee, V. A novel cnn-gru-lstm based deep learning model for accurate traffic prediction. *Discover Computing* **28**, 38 (2025).

[31] Boddu, Y. & Manimaran, A. Design of an iterative method for time series forecasting using temporal attention and hybrid deep learning architectures. *IEEE Access*10.1109/ACCESS.2025.3538577 (2025).

[32] Zheng, W. & Hu, J. Multivariate time series prediction based on temporal change information learning method. *IEEE Trans. Neural Netw. Learn. Syst.* **34**, 7034–7048 (2022). 10.1109/TNNLS.2021.3137178 34982703

[33] Wu, B., Wang, L. & Zeng, Y.-R. Interpretable wind speed prediction with multivariate time series and temporal fusion transformers. *Energy* **252**, 123990 (2022).

[34] Xu, Y., Shen, S.-Q., He, Y.-L. & Zhu, Q.-X. A novel hybrid method integrating ica-pca with relevant vector machine for multivariate process monitoring. *IEEE Trans. Control Syst. Technol.* **27**, 1780–1787 (2018).

[35] Chen, S., Chang, C.-I. & Li, X. Component decomposition analysis for hyperspectral anomaly detection. *IEEE Trans. Geosci. Remote Sens.* **60**, 1–22 (2021).

[36] Boddu, Y., Manimaran, A., Arunkumar, B. & Ramkumar, D. Design of an iterative dual metaheuristic VARMAX model enhancing efficiency of time series predictions. *IEEE Access*10.1109/ACCESS.2024.3454540 (2024).

[37] Patil, M., Vyawahare, V. & Birajdar, G. *Intelligent Computing and Big Data Analytics: First International Conference, ICICBDA 2024, Navi Mumbai, India, June 15–16, 2024, Proceedings, Part-I* Vol. 2234 (Springer Nature, 2024).

